# Spectrum and signals of medication-associated cognitive disorder: a comprehensive disproportionality analysis with cross-database validation

**DOI:** 10.3389/fphar.2026.1762761

**Published:** 2026-04-10

**Authors:** Hao Wen, Yajie Dong, Ruiting Wang

**Affiliations:** 1 Institute of Traditional Chinese Medicine/Hebei Key Laboratory of Nerve Injury and Repair, Chengde Medical University, Chengde, China; 2 Department of Pathophysiology, Chengde Medical University, Chengde, China; 3 Hebei Key Laboratory of Nerve Injury and Repair/Department of Pharmacology, Chengde Medical University, Chengde, China

**Keywords:** adverse effects, cognitive disorder, disproportionality analysis, FAERS database, pharmacovigilance

## Abstract

**Objective:**

The clinical risk of cognitive disorders linked to various drugs is not well-defined. This study aimed to identify medications with notable signals for drug-related cognitive disorders and evaluate whether their US Food and Drug Administration (FDA)-approved labels include relevant safety warnings.

**Methods:**

A retrospective disproportionality analysis using FDA Adverse Event Reporting System data (2004–2024) employed four algorithms—Reporting Odds Ratio (ROR), Proportional Reporting Ratio (PRR), Bayesian Confidence Propagation Neural Network (BCPNN) and Multi-Item Gamma Poisson Shrinker (MGPS)—for signal detection. To confirm the results, a cross-database consistency check was performed with the Japanese Adverse Drug Event Report (JADER) and World Health Organization (WHO) VigiAccess databases.

**Results:**

An analysis of 41,775 reports on drug-related cognitive disorders found significant signals for 50 medications using four algorithms. Notably, 74% of these drugs, including finasteride, diltiazem, and carbidopa/levodopa, lacked cognitive disorder warnings in FDA labels. Reporting patterns were classified into early or random failure types. Subgroup analyses showed certain drugs were disproportionately reported in vulnerable groups, such as antiepileptics in children and neurologic agents in seniors. The U.S. had the most reports. Multivariate analysis identified 43 factors linked to higher reporting odds, including conditions like depression and the use of specific drugs. Cross-database validation confirmed consistent signals for 92% of primary drug-event pairs.

**Conclusion:**

This pharmacovigilance analysis uncovers significant, previously unrecognized signals of drug-related cognitive disorders in various medications, most lacking label warnings. These findings highlight the need for further research, potential label updates, and increased clinical awareness, particularly in high-risk groups. Importantly, these results indicate statistical associations, not causal links, between the drugs and cognitive disorders.

## 1 Introduction

Cognitive disorder, characterized by acquired impairments in higher-order mental processes, represents a clinically significant condition that markedly hinders an individual’s capacity to conduct activities of daily living, sustain occupational performance, and participate in social interactions (Marshall et al., 2019). From a neuropsychological standpoint, cognition encompasses a multifaceted array of mental processes, including information acquisition, memory consolidation, executive functioning, attentional regulation, linguistic processing, and problem-solving capabilities (Pérez-Blanco and Rodríguez-Salgado, 2022). These cognitive functions are orchestrated by complex neural networks that enable the encoding, storage, retrieval, and integration of information across various brain regions (Iinuma et al., 2022).

In pathological terms, cognitive disorders are characterized by impaired higher cortical functions, particularly impacting learning, memory, and executive functioning, and are often accompanied by neurobehavioral symptoms such as aphasia, apraxia, agnosia, or affective disturbances (Ehrhardt et al., 2018). These disorders result from complex neurobiological dysregulation, with primary mechanisms including neurodegeneration driven by pathological protein aggregation (e.g., amyloid-β, tau) (Kim et al., 2025), synaptic dysfunction and neurotransmitter deficits (e.g., cholinergic, glutamatergic) (Wang et al., 2025), chronic neuroinflammation mediated by glial activation (Wang et al., 2022), mitochondrial impairment leading to oxidative stress and bioenergetic failure, and cerebrovascular pathology (Busby et al., 2025; Zhang et al., 2024). These processes interact synergistically, and when combined with genetic predispositions and disrupted neural network connectivity, ultimately impair cognitive circuitry. In the context of pharmacology, cognitive adverse events have been reported with numerous medications and represent a critical domain for post-marketing safety surveillance (Yu et al., 2021). Despite its clinical significance, contemporary drug development protocols frequently lack comprehensive cognitive assessment measures, which may result in the oversight of subtle yet clinically pertinent cognitive effects of therapeutic agents.

Certain pharmacological agents are well-documented to have cognitive disorder effects, prompting updates to labeling regarding their sedative properties. However, the complete range of medications associated with disproportionate reports of cognitive disorders remains inadequately defined. Benzodiazepines and Z-class drugs, like zolpidem, are recognized for inducing sedation and memory impairment, particularly among older adults. Despite their prevalent use, these agents are associated with cognitive decline, with the geriatric population being most vulnerable (Tseng et al., 2020). Clinical studies have demonstrated that benzodiazepines and Z-class drugs impair memory by disrupting memory consolidation, affecting both the formation and retrieval of memories (Zanin et al., 2013). Prolonged use can lead to a global cognitive decline, impacting attention, processing speed and working memory (Stranks and Crowe, 2014). The association between benzodiazepine or Z-drug use and dementia remains debated. Some studies suggest that residual confounding and protopathic bias may partly explain the observed link (Friesen et al., 2025), while others emphasize that half-life (Legrand et al., 2025) and cumulative exposure (Vakili et al., 2025) are important modifiers. A recent meta-analysis concluded that prolonged use of GABAergic drugs is associated with an increased dementia risk, but causality has not been firmly established (Vakili et al., 2025). Anticholinergic medications, which include certain antihistamines, tricyclic antidepressants, and treatments for overactive bladder, have been associated with delirium and long-term cognitive decline (Kotochinsky et al., 2025). Additionally, specific antiepileptic drug (such as topiramate and phenobarbital) (Li et al., 2025), corticosteroids, and chemotherapeutic agents (such as methotrexate) are associated with cognitive dysfunction through various mechanisms (Reimers et al., 2025). This gap in knowledge is particularly concerning, as cognitive disorders can significantly affect patient safety, treatment adherence, and overall quality of life in diverse clinical contexts (Seccomandi et al., 2022). Therefore, the systematic identification of medications with a disproportionate incidence of cognitive adverse events is crucial for pharmacovigilance and the assessment of treatment risk.

The FDA Adverse Event Reporting System (FAERS) is an extensive, publicly accessible pharmacovigilance database that functions as a repository for post-marketing surveillance drug reactions (ADRs), product quality issues, and medication error reports related to FDA-approved therapeutic agents (Zhou et al., 2023; Yin et al., 2022). This system employs a dual reporting mechanism, whereby pharmaceutical manufacturers are required to submit documentation of adverse event, and healthcare providers and consumers globally are encouraged to submit voluntary reports (Anand et al., 2019).

Recent analyses utilizing data from the FAERS database indicate a trend towards the adoption of more standardized and diverse methodologies in drug safety research, particularly concerning neurocognitive and psychiatric adverse events. Disproportionality analysis, employing the reporting odds ratio (ROR) and proportional reporting ratio (PRR), serves as the principal statistical technique for detecting drug risk signals (Yang et al., 2023). This method has been successfully applied to investigate a range of cognitive and communication disorders associated with various drug classes, thereby affirming its utility in neuropsychological pharmacovigilance (Lin J. et al., 2025; Wang Y. et al., 2024). Zhao et al. employed integrated ROR and BCPNN analyses on everolimus-related reports from the FAER, confirming established risks such as pneumonitis and stomatitis, while also identifying a novel risk, lymphocele (Zhao et al., 2024). Similarly, Zhong et al. utilized the PRR with multivariate correction to compare vinorelbine and vincristine, discovering that vinorelbine exhibited a stronger signal for hematologic disorders, whereas vincristine was linked to vascular disorders (Zhong et al., 2024). These studies underscore the significance of FAERS-based analyses in enhancing post-marketing surveillance and developing personalized treatment strategies. The rigorous methodologies and demonstrated sensitivity of these algorithms robustly endorse their application in systematic evaluation of drug-associated cognitive disorders—a domain characterized by fragmented safety evidence and frequent lack of label warnings.

The fundamental importance of FAERS in pharmacovigilance arises from the intrinsic limitations associated with pre-marketing clinical trials. These trials are generally characterized by controlled study designs, stringent inclusion and exclusion criteria, restricted participant populations, and brief observation on periods (Zou F. et al., 2024; Godfrey et al., 2025). Such limitations highlight the critical needs for post-marketing surveillance systems, wherein spontaneous reporting mechanisms are indispensable for detecting potential safety signals and assessing suspected adverse drug events within real-world clinical environments.

This pharmacovigilance study systematically analyzed the most recent FAERS data to assess drug-associated cognitive disorder events over an extensive 20-year surveillance period (Q1 2004 to Q4 2024). The primary objective was to identify significant disproportionality signals for cognitive disorders across various therapeutic agents.

## 2 Methods

### 2.1 Data sources and collection

This study employed the Medical Dictionary for Regulatory Activities (MedDRA) classification system for adverse drug event (ADE) categorization, using System Organ Class (SOC) as the highest-level classification and Preferred Terms (PTs) for standardized event documentation. Consistent with standard pharmacovigilance practices, all reported events were mapped to relevant PTs in the MedDRA (version 27.1) to enable quantitative analysis (Wen et al., 2024). The analytical framework first involved extracting all PTs from the MedDRA database, followed by application of a frequency threshold (n > 3 occurrences) within the FAERS database to ensure statistical robustness (Lin et al., 2023).

In alignment with FDA guidelines for the management of pharmacovigilance data, the most recent FDA decision date (FDA DT) was employed to temporally categorize reports (Zou S. et al., 2024). In instances where entries sharing identical CASEID and FDA DT values, the record possessing the highest PRIMARYID was retained to remove duplicate submissions originating from multiple sources. The research protocol was designed to collect and analyze clinically pertinent parameters associated with ADEs related to cognitive disorders. This included demographic variables (age, gender), reporting metrics (source, country, year), and clinical outcome data.

**FIGURE 1:**
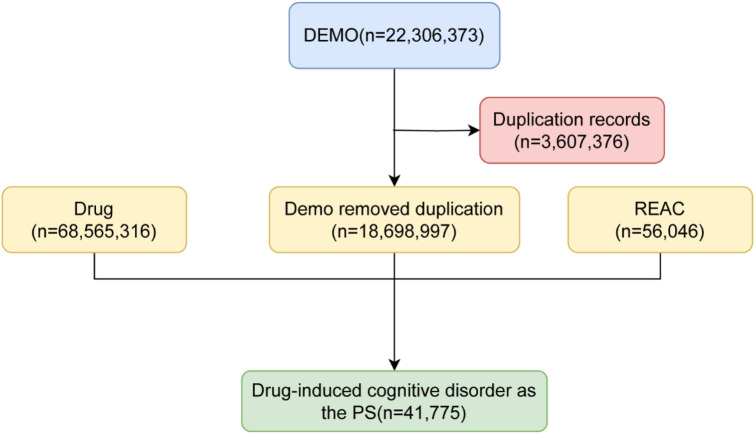
The flow diagram of selecting cognitive disorder associated with drug used from FAERS database.

Reports of cognitive disorder adverse events were extracted from the FAERS database from the first quarter of 2004 to the fourth quarter of 2024. Cases that were unrelated to pharmacotherapy, contained incomplete or unassessable data, or were duplicate entries were excluded from the analysis. Reports that identified “cognitive disorder” (PT code: 10057668) as a target ADR were selected to capture drug-induced events. To mitigate under-ascertainment bias and validate our primary findings, a sensitivity analysis was conducted using an expanded case definition. This definition comprised a composite “cognitive disorder” endpoint that included four MedDRA PTs: Cognitive disorder (PT code: 10057668), Memory impairment (PT code: 10027175), Confusional state (PT code: 10010305) and Disturbance in attention (PT code: 10013496). For relevance, cases that were not drug-induced, reports that were unassessable, duplicates and incidents related to overdose were excluded from the analysis (Figure 1). Figure 1 interpreted to clarify the rigorous inclusion/exclusion criteria for FAERS data curation, including the removal of duplicates, non-drug-induced cases, and incomplete records; this step is highlighted to justify the robustness of the final 41,775 cognitive disorder reports and mitigate under-ascertainment bias. In the disproportionate analysis, only those drugs identified as “primary suspicion (PS)” were considered for the final analysis. Reports in which the drug was categorized as “secondary suspicion (SS),” “combination drug” or “interacting drug” were excluded to enhance the causal inference between the drug and the reported cognitive disorder event. This approach resulted in a final dataset comprising patient demographics (age, sex), reporting details (year, country), and information on the suspect medication. The application of these validated pharmacovigilance techniques supports the generation of reliable safety signals and improves the detection of potential cognitive disorder effects associated with pharmacological interventions.

### 2.2 Statistical analysis

This study utilized a comprehensive analytical framework integrating descriptive statistics with quantitative pharmacovigilance methodologies to assess adverse events related to cognitive disorders (Ye et al., 2024). Descriptive statistical analyses were systematically performed to delineate the demographic and clinical characteristics of reported ADR (Yang et al., 2024). For signal detection, several validated disproportionality measures, including the ROR and PRR, were employed to identify statistically significant ADR reporting patterns that surpassed anticipated background frequencies (Yao et al., 2025).

The disproportionality analysis employed four well-established data mining algorithms—ROR, PRR, Bayesian Confidence Propagation Neural Network (BCPNN) and Multi-item Gamma Poisson Shrinker (MGPS)—to identify potential adverse drug reaction signals by quantifying statistical associations within the FAERS. The results of the safety signal detection are presented as point estimates accompanied by their respective confidence intervals: ROR (ROR and 95% CI), PRR (PRR and 95% CI), BCPNN (IC and IC025), and MGPS (EBGM and EBGM025). Each method generates distinct signal metrics: ROR (lower 95% CI > 1), PRR (ratio ≥2 combined with χ^2^ ≥ 4), BCPNN (information component IC025 > 0), and MGPS (EBGM025 > 2.0). The analyses required a minimum of three case reports per drug-event combination and utilized the Bonferroni correction to control false positives. All analyses were conducted using validated pharmacovigilance software—PhViD for ROR, PRR, and BCPNN, and openEBGM for EBGM—in accordance with the methodological standards of the European Medicines Agency (EMA) and the FDA (Supplementary Tables S1, S2).

A drug-event combination was classified as signal-positive only if it satisfied the thresholds across all four disproportionality algorithms (ROR, PRR, BCPNN, and MGPS). This methodological approach mitigates the dependency on a singular analytical technique and reduces the incidence of false positives. The Bonferroni correction was applied to adjust for multiple comparisons across the extensive array of drug-event pairs analyzed. Although not commonly employed in routine pharmacovigilance, this correction was deemed suitable for this large-scale exploratory investigation due to the clinical significance of cognitive disorder outcomes and the imperative to minimize false positives. The conventional alpha level of 0.05 was divided by the number of drug-event combinations tested, thereby establishing a more stringent threshold for statistical significance and enhancing the robustness of the findings.

The temporal interval between the initiation of medication and the onset of cognitive disorder symptoms, referred to as the time-to-onset (TTO), was analyzed using both descriptive and inferential statistical methods. To maintain data integrity, records with implausible or inconsistent temporal information were systematically excluded. These exclusions included cases where: (1) the drug initiation date (start_dt) occurred after the adverse event onset date (event_dt); (2) the adverse event onset date (event_dt) was later than the FDA report receipt date (fda_dt); (3) the patient birth date (birth_dt) was subsequent to the adverse event onset date (event_dt); (4) invalid date formats or impossible date values were present (e.g., 0000-00-00, 9999-99-99, month > 12, day > 31). These exclusions were executed programmatically. To characterize the distribution patterns of TTO, robust statistical measures were employed, including median values, interquartile ranges (IQRs), and parameters of the Weibull distribution.

The Weibull distribution model, characterized by its scale parameter (α) and shape parameter (β), was utilized to assess the temporal dynamics of risk associated with drug-associated cognitive disorders (Nagura et al., 2025; Liu et al., 2026). The scale parameter (α) serves as the characteristic life parameter, whereas the shape parameter (β) determines the trend of the risk profile over time, indicating whether it is increasing (β > 1), constant (β = 1) or decreasing (β < 1). Analysis of shape parameter (β) revealed two distinct patterns: an early failure pattern, where β < 1 (95% CI excluding 1), signifying a declining incidence of adverse events over time; and a random failure pattern, where β ≈ 1 (95% CI including 1), indicating a constant probability of AE occurrence over time. This parametric survival analysis approach offers significant insights into the temporal characteristics and risk progression patterns of cognitive disorder associated with drug use.

To conduct a more comprehensive analysis of the effects of stratification on drug-associated cognitive disorders, participants were categorized based on gender (female, male), age groups (≤18, 19 to <45, 45 to <65, ≥65 years) and weight categories (<50, 50 to ≤100, >100 kg). Furthermore, the study focused on the five countries reporting the highest incidence of drug-associated cognitive disorders: the United States, Canada, the United Kingdom, France and Germany.

### 2.3 Variables impacting cognitive disorder induced by medication

Potential risk factors for drug-associated cognitive disorders were systematically evaluated, encompassing variables such as age, sex, weight, type of reporter, the top 20 comorbidities, and the top 30 concomitant medications reported in qualifying adverse events. Associations were initially assessed using univariate logistic regression, followed by multivariate logistic regression to refine the analysis. Odds ratios (ORs) were computed for each factor. Covariates included in the final multivariate model were selected through stepwise regression, employing a significance threshold of P < 0.05. To mitigate potential multicollinearity among the risk factors, the variance inflation factor (VIF) was calculated for each variable in the final model, with a VIF threshold of less than 5 to ensure acceptable levels of collinearity. The drugs incorporated into the regression analysis were those identified as signal-positive in the initial disproportionality assessment (Table 3), with an emphasis on pertinent pharmacological associations. We recognize the limitations inherent in applying regression models to SRS data, such as the lack of a defined denominator, uncertain drug exposure and potential confounding by indication. Consequently, the results of logistic regression are interpreted as identifying statistical associations and generating hypotheses for further investigation, rather than establishing definitive causal risk factors.

### 2.4 Cross-database validation

To assess the robustness of signals derived from the FAERS, reports pertaining to the PT cognitive disorder (PT code 10057668) were extracted from the Japanese Adverse Drug Event Report (JADER) database for the period spanning 2004 to 2024, thereby aligning with the FAERS research timeline. JADER employed an identical statistical framework and adhered to the same signal detection standards as FAERS, utilizing the four disproportionate analysis (ROR, PRR, BCPNN and MGPS) with consistent algorithm and threshold calculation. The data underwent rigorous cleaning procedures, including the removal of duplicates entries and the exclusion of reports involving non-primary suspected drugs.

The validation process was conducted on the 50 pharmaceuticals with the greatest number of cognitive disorder reports in the FAERS database. For the World Health Organization VigiAccess database, data as of December 2024, was queried to ascertain the total number of cognitive disorder reports associated with these 50 drugs. The VigiAccess data served exclusively as supplementary verification to compare reporting frequencies across various pharmacovigilance systems and was not utilized for statistical signal verification.

## 3 Result

### 3.1 Demographic and clinical profiles of cognitive disorder-related adverse drug reactions

An analysis of FAERS database revealed a cumulative total of 56,046 adverse event reports related to cognitive disorders up to Q4 2024, with 41,775 cases specifically documented under the preferred term cognitive disorder (Table 1). A longitudinal analysis indicated a consistent annual increase in reports of cognitive disorder-related events from Q1 2004 to Q4 2024. Geospatial distribution analysis identified the United States (n = 20,655), Canada (n = 2,423), the United Kingdom (n = 2,316), France (n = 1,419), and Germany (n = 1,054) as the top five reporting countries in terms of report frequency.

**TABLE 1:** Demographic Distribution of cognitive disorder associated with drug used Reports (2004–2024).

Variables	N (%)
Year	
2004	299 (0.72)
2005	400 (0.96)
2006	453 (1.08)
2007	540 (1.29)
2008	545 (1.30)
2009	578 (1.38)
2010	1,302 (3.12)
2011	1,517 (3.63)
2012	1,670 (4.00)
2013	2,313 (5.54)
2014	2,141 (5.13)
2015	2,600 (6.22)
2016	2,593 (6.21)
2017	2,671 (6.39)
2018	3,018 (7.22)
2019	2,811 (6.73)
2020	3,346 (8.01)
2021	3,432 (8.22)
2022	3,289 (7.87)
2023	3,042 (7.28)
2024	3,213 (7.69)
Sex	
Female	23432 (56.09)
Male	15389 (36.84)
Unknown	2,952 (7.07)
Age, years	57.00 (43.00, 71.00)
<=18	1,087 (2.60)
19∼45	5,958 (14.26)
45∼65	9,426 (22.57)
>=65	9,643 (23.08)
Unknow	15658 (37.48)
Weight, kg	73.00 (60.60, 86.26)
<50	895 (2.14)
50∼100	8,637 (20.68)
>=100	1,240 (2.97)
Unknow	31001 (74.21)
Reporter	
Consumer	20249 (48.47)
Physician	9,054 (21.67)
Pharmacist	5,189 (12.42)
Other health-professional	4,712 (11.28)
Unknown	1,601 (3.83)
Lawyer	946 (2.26)
Registered nurse	22 (0.05)
Reported countries	
United States	20655 (49.45)
Other	8,754 (20.96)
Canada	2,423 (5.80)
United Kingdom	2,316 (5.54)
France	1,419 (3.40)
Germany	1,054 (2.52)
Netherlands	724 (1.73)
Japan	691 (1.65)
Italy	568 (1.36)
Spain	505 (1.21)
Australia	449 (1.07)
Denmark	250 (0.60)
China	245 (0.59)
Brazil	211 (0.51)
Sweden	202 (0.48)
Poland	163 (0.39)
Switzerland	153 (0.37)
Belgium	144 (0.34)
Portugal	120 (0.29)
Israel	115 (0.28)
Norway	113 (0.27)
Czechia	92 (0.22)
India	71 (0.17)
Turkey	62 (0.15)
Korea, South	61 (0.15)
Austria	56 (0.13)
Argentina	54 (0.13)
Mexico	53 (0.13)
Finland	50 (0.12)
Outcomes	
Other serious	21220 (48.98)
Hospitalization	13192 (30.45)
Disability	4,434 (10.23)
Life threatening	1915 (4.42)
Death	1,658 (3.83)
Congenital anomaly	537 (1.24)
Required intervention to prevent permanent impairment/damage	366 (0.84)
Time-to-onset, days	60.00 (2.00, 498.75)
<2	2,449 (9.53)
2∼5	443 (1.72)
5∼7	185 (0.72)
7∼14	454 (1.77)
14∼28	615 (2.39)
>=28	5,770 (22.45)
Unknow	15791 (61.43)

In the cohort of patients experiencing adverse events, the median age was 57.0 years (IQR: 43.0–71.0 years), median body weight was 73.0 kg (IQR: 60.6–86.3 kg), and median TTO was 60.0 days (IQR: 2.0–498.8 days). The age distribution was as follows: patients aged 18 years or younger constituted 2.60% (n = 1,087); those aged 19–44 years represented 14.26% (n = 5,958); individuals aged 45–64 years accounted for 22.57% (n = 9,426); those aged 65 years and older comprised 23.08% (n = 9,643) and patients with unknown age made up 37.48% (n = 15,658). Regarding body weight, 2.14% (n = 895) of patients weighed less than 50 kg, 20.68% (n = 8,637) weighed between 50 and 100 kg, 2.97% (n = 1,240) weighed 100 kg or more, and 74.21% (n = 31,001) had unknown weight. The distribution of TTO was as follows: less than 2 days in 9.53% (n = 2,449) of cases, 2–5 days in 1.72% (n = 443), 5–7 days in 0.72% (n = 185), 7–14 days in 1.77% (n = 454), 14–28 days in 2.39% (n = 615), 28 days or more in 22.45% (n = 5,770), and unknown TTO in 61.43% (n = 15,791) of cases.

A pharmacological analysis of the 50 medications most frequently associated with cognitive disorders identified natalizumab (n = 3,254), interferon beta-1a (n = 2,124), dimethyl fumarate (n = 1,440), finasteride (n = 1,370) and carbidopa/levodopa (n = 1,245) as the top five agents. Of note, 74% (n = 37) of these medications do not currently include cognitive disorder warnings in their prescribing information. However, it is important to acknowledge that for drugs such as carbidopa/levodopa—which is used almost exclusively in Parkinson’s disease—reported cognitive adverse events may reflect, at least in part, the natural progression of the underlying neurodegenerative condition rather than a direct drug effect. This complex interplay between disease pathology and pharmacological exposure must be considered when interpreting these findings. A comprehensive summary of the warning status for each high-risk drug is provided in Table 2.

**TABLE 2:** Quantitative analysis of medication-related cognitive disorder effects: Identification of top 50 high-risk therapeutic agents.

Drug	Case reports	ROR (95% CI)	PRR (95% CI)	IC (IC025)	EBGM (EBGM05)	P.value	P.adj	Cognitive disorder warning
Finasteride	1,370	54.09 (51.1, 57.25)	48.52 (45.75, 51.46)	5.55 (5.47)	46.96 (44.78)	<0.001	<0.001	No
Oxcarbazepine	151	31.34 (26.57, 36.98)	29.36 (25.1, 34.34)	4.87 (4.63)	29.26 (25.48)	<0.001	<0.001	Yes
Lorlatinib	115	13.56 (11.26, 16.32)	13.19 (11.06, 15.73)	3.72 (3.45)	13.15 (11.26)	<0.001	<0.001	Yes
Diltiazem hydrochloride	44	12.53 (9.28, 16.91)	12.21 (9.1, 16.38)	3.61 (3.18)	12.2 (9.5)	<0.001	<0.001	No
Valproate sodium	511	11.26 (10.31, 12.3)	11.01 (10.18, 11.91)	3.45 (3.32)	10.89 (10.12)	<0.001	<0.001	Yes
Carbidopa/levodopa	1,245	10.94 (10.33, 11.58)	10.71 (10.1, 11.36)	3.38 (3.3)	10.42 (9.93)	<0.001	<0.001	No
Avapritinib	192	10.8 (9.36, 12.47)	10.57 (9.21, 12.12)	3.4 (3.19)	10.53 (9.34)	<0.001	<0.001	Yes
Diroximel fumarate	186	9.64 (8.33, 11.15)	9.46 (8.25, 10.85)	3.24 (3.03)	9.42 (8.34)	<0.001	<0.001	No
Natalizumab	3,254	9.48 (9.15, 9.83)	9.32 (8.96, 9.69)	3.12 (3.06)	8.67 (8.41)	<0.001	<0.001	No
Nitroglycerin	78	9.08 (7.25, 11.36)	8.92 (7.19, 11.07)	3.15 (2.83)	8.9 (7.38)	<0.001	<0.001	No
Topiramate	488	8.29 (7.57, 9.07)	8.15 (7.39, 8.99)	3.01 (2.88)	8.07 (7.49)	<0.001	<0.001	Yes
Donepezil	162	8.1 (6.93, 9.47)	7.98 (6.82, 9.33)	2.99 (2.77)	7.95 (6.98)	<0.001	<0.001	No
Divalproex sodium	210	7.45 (6.5, 8.54)	7.35 (6.41, 8.43)	2.87 (2.67)	7.31 (6.52)	<0.001	<0.001	Yes
Haloperidol	180	6.79 (5.86, 7.87)	6.7 (5.84, 7.69)	2.74 (2.53)	6.68 (5.9)	<0.001	<0.001	Yes
Dimethyl fumarate	1,440	6.21 (5.89, 6.55)	6.14 (5.79, 6.51)	2.58 (2.5)	5.96 (5.7)	<0.001	<0.001	No
Teriflunomide	531	6.19 (5.68, 6.75)	6.12 (5.66, 6.62)	2.6 (2.47)	6.06 (5.64)	<0.001	<0.001	No
Interferon beta-1a	1984	5.99 (5.72, 6.27)	5.93 (5.7, 6.17)	2.51 (2.44)	5.69 (5.48)	<0.001	<0.001	No
Fingolimod hydrochloride	1,001	5.79 (5.43, 6.16)	5.73 (5.4, 6.08)	2.49 (2.4)	5.61 (5.32)	<0.001	<0.001	No
Peginterferon beta-1a	140	5.58 (4.72, 6.6)	5.53 (4.73, 6.47)	2.46 (2.22)	5.51 (4.79)	<0.001	<0.001	No
Rivastigmine	177	5.51 (4.75, 6.4)	5.46 (4.76, 6.26)	2.44 (2.23)	5.44 (4.81)	<0.001	<0.001	No
Clonazepam	224	5.17 (4.53, 5.9)	5.12 (4.46, 5.87)	2.35 (2.16)	5.1 (4.56)	<0.001	<0.001	No
Lansoprazole	47	5.09 (3.82, 6.79)	5.04 (3.76, 6.76)	2.33 (1.92)	5.04 (3.96)	<0.001	<0.001	No
Carbamazepine	234	4.97 (4.36, 5.65)	4.92 (4.29, 5.64)	2.29 (2.11)	4.9 (4.4)	<0.001	<0.001	No
Paroxetine	38	4.86 (3.53, 6.69)	4.82 (3.52, 6.6)	2.27 (1.81)	4.81 (3.68)	<0.001	<0.001	Yes
Siponimod	81	4.63 (3.72, 5.76)	4.59 (3.7, 5.69)	2.2 (1.88)	4.59 (3.82)	<0.001	<0.001	Yes
Alemtuzumab	116	4.59 (3.82, 5.51)	4.55 (3.81, 5.43)	2.18 (1.92)	4.54 (3.9)	<0.001	<0.001	No
Pimavanserin tartrate	373	4.47 (4.03, 4.95)	4.43 (4.02, 4.89)	2.14 (1.99)	4.4 (4.04)	<0.001	<0.001	No
Levetiracetam	471	4.03 (3.68, 4.41)	4 (3.63, 4.41)	1.99 (1.86)	3.97 (3.68)	<0.001	<0.001	Yes
Lacosamide	40	3.99 (2.92, 5.45)	3.97 (2.9, 5.43)	1.99 (1.54)	3.96 (3.05)	<0.001	<0.001	No
Lorazepam	45	3.88 (2.89, 5.21)	3.86 (2.88, 5.18)	1.95 (1.53)	3.85 (3.02)	<0.001	<0.001	Yes
Oxycodone	41	3.43 (2.52, 4.66)	3.41 (2.49, 4.67)	1.77 (1.33)	3.4 (2.63)	<0.001	<0.001	No
Ocrelizumab	356	3.16 (2.85, 3.51)	3.14 (2.85, 3.46)	1.64 (1.49)	3.13 (2.86)	<0.001	<0.001	No
Dalfampridine	406	3.12 (2.83, 3.44)	3.11 (2.82, 3.43)	1.63 (1.48)	3.08 (2.84)	<0.001	<0.001	No
Escitalopram oxalate	327	3.11 (2.79, 3.47)	3.09 (2.75, 3.48)	1.62 (1.46)	3.08 (2.81)	<0.001	<0.001	No
Tramadol	40	2.99 (2.19, 4.08)	2.98 (2.18, 4.08)	1.57 (1.13)	2.98 (2.29)	<0.001	<0.001	No
Olanzapine	326	2.96 (2.66, 3.31)	2.95 (2.62, 3.32)	1.55 (1.4)	2.94 (2.68)	<0.001	<0.001	No
Lamotrigine	341	2.95 (2.65, 3.28)	2.94 (2.67, 3.24)	1.55 (1.39)	2.92 (2.67)	<0.001	<0.001	No
Ofatumumab	174	2.95 (2.54, 3.42)	2.94 (2.51, 3.44)	1.55 (1.33)	2.93 (2.58)	<0.001	<0.001	No
Gabapentin	434	2.94 (2.67, 3.23)	2.92 (2.65, 3.22)	1.54 (1.4)	2.9 (2.68)	<0.001	<0.001	No
Niraparib	129	2.86 (2.4, 3.4)	2.84 (2.38, 3.39)	1.51 (1.26)	2.84 (2.46)	<0.001	<0.001	No
Ixazomib	46	2.76 (2.06, 3.68)	2.74 (2.04, 3.68)	1.46 (1.04)	2.74 (2.15)	<0.001	<0.001	No
Zolpidem	46	2.71 (2.03, 3.62)	2.7 (2.01, 3.62)	1.43 (1.02)	2.7 (2.11)	<0.001	<0.001	No
Phenytoin	99	2.7 (2.22, 3.3)	2.69 (2.21, 3.27)	1.43 (1.14)	2.69 (2.28)	<0.001	<0.001	Yes
Vimpat	44	2.69 (2, 3.62)	2.68 (2, 3.6)	1.42 (1)	2.68 (2.09)	<0.001	<0.001	No
Mirtazapine	109	2.62 (2.17, 3.17)	2.61 (2.15, 3.18)	1.38 (1.11)	2.61 (2.23)	<0.001	<0.001	No
Sertraline hydrochloride	295	2.56 (2.28, 2.87)	2.55 (2.27, 2.87)	1.34 (1.18)	2.54 (2.31)	<0.001	<0.001	No
Fluoxetine	141	2.48 (2.1, 2.93)	2.48 (2.12, 2.9)	1.3 (1.07)	2.47 (2.15)	<0.001	<0.001	Yes
Venlafaxine hydrochloride	265	2.43 (2.15, 2.74)	2.42 (2.15, 2.72)	1.27 (1.09)	2.41 (2.18)	<0.001	<0.001	No
Interferon beta-1b	128	2.43 (2.04, 2.89)	2.42 (2.03, 2.89)	1.27 (1.02)	2.42 (2.09)	<0.001	<0.001	No
Duloxetine hydrochloride	252	2.41 (2.13, 2.73)	2.4 (2.13, 2.7)	1.26 (1.08)	2.39 (2.16)	<0.001	<0.001	No

### 3.2 Pharmacovigilance analysis of cognitive disorder-related adverse drug reactions

A disproportionality analysis identified significant pharmacovigilance findings related to adverse drug reactions associated with cognitive disorders. Among the 50 medications exhibiting the most pronounced safety signals, 74% (n = 37) did not include warnings about cognitive disorders in their prescribing information, indicating previously unidentified safety concerns. The agents with the strongest disproportionality signals, ranked by signal strength, were as follow: finasteride [n = 1,370, ROR 54.09 (51.10, 57.25), PRR 48.52 (45.75, 51.46), IC 5.55 (5.47), EBGM 46.96 (44.78)], oxcarbazepine [n = 151, ROR 31.34 (26.57, 36.98), PRR 29.36 (25.1, 34.34), IC 4.87 (4.63), EBGM 29.26 (25.48)], valproate sodium [n = 511, ROR 11.26 (10.31, 12.3), PRR 11.01 (10.18, 11.91), IC 3.45 (3.32), EBGM 10.89 (10.12)], carbidopa/levodopa [n = 1,245, ROR 10.94 (10.33, 11.58), PRR 10.71 (10.10, 11.36), IC 3.38 (3.3), EBGM 10.42 (9.93)] and diroximel fumarate [n = 186, ROR 9.64 (8.33, 11.15), PRR 9.46 (8.25, 10.85), IC 3.24 (3.03), EBGM 9.42 (8.34)].

**FIGURE 2:**
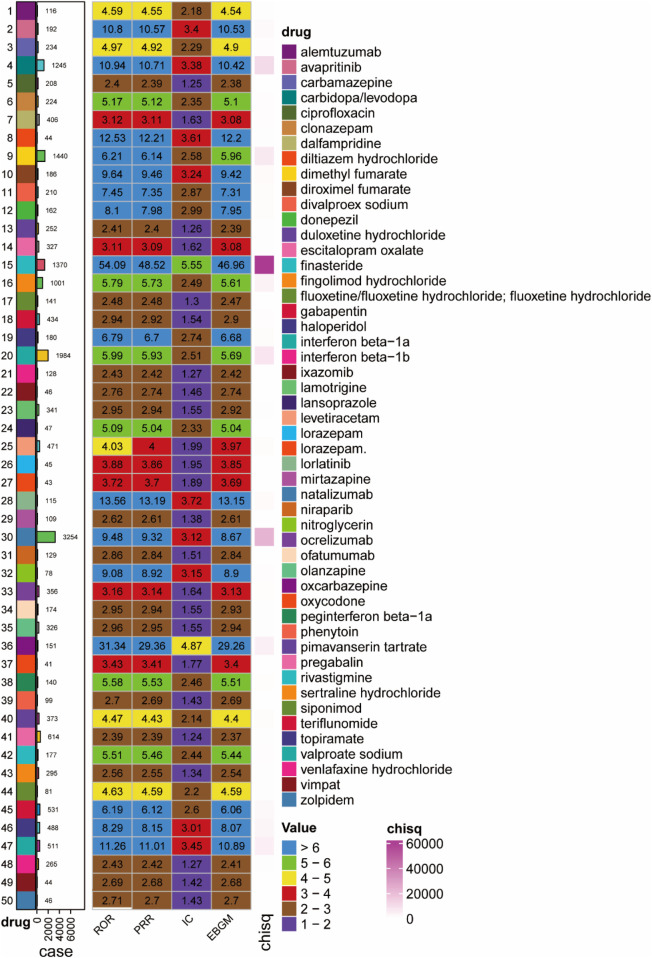
Quantitative analysis of medication-related cognitive disorder effects: Identification of top 50 high-risk therapeutic agents.

Several drugs with the highest volume of cognitive disorder reports also lacked corresponding label warnings, including natalizumab [n = 3,254, ROR 9.48 (9.15, 9.83), PRR 9.32 (8.96, 9.69); IC 3.12 (3.06), EBGM 8.67 (8.41)], interferon beta-1α [n = 1984, ROR 5.99 (5.72, 6.27), PRR 5.93 (5.7, 6.17), IC 2.51 (2.44), EBGM 5.69 (5.48)], dimethyl fumarate [n = 1,440, ROR 6.21 (5.89, 6.55), PRR 6.14 (5.79, 6.51), IC 2.58 (2.5), EBGM 5.96 (5.7)], finasteride [n = 1,370, ROR 54.09 (51.1, 57.25), PRR 48.52 (45.75, 51.46), IC 5.55 (5.47), EBGM 46.96 (44.78)] and carbidopa/levodopa combination [n = 1,245, ROR 10.94 (10.33, 11.58), PRR 10.71 (10.1, 11.36), IC 3.38 (3.3), EBGM 10.42 (9.93)]. A comprehensive analysis of adverse events related to cognitive disorders is depicted in Figure 2 and Table 2, which detail the temporal patterns and demographic distributions of these pharmacovigilance findings. Consistent with the summary of warning status (Table 2), drugs such as finasteride, natalizumab, and carbidopa/levodopa demonstrated significant safety signals but did not include corresponding cognitive disorder warnings in their labeling. Figure 2 interpreted to emphasize the hierarchical signal strength of the 50 drugs (e.g., finasteride with the highest ROR/PRR/IC/EBGM values); the figure’s visual comparison of four disproportionality algorithms is noted to validate the consistency of the identified safety signals.

### 3.3 Temporal association between pharmacological exposure and cognitive disorder onset

**FIGURE 3:**
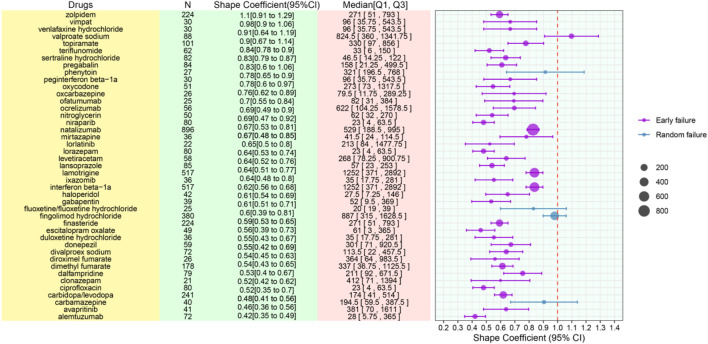
Time-to-onset based categorization framework for drug-induced adverse effects.

For the 50 pharmacological agents most strongly associated with cognitive disorders, temporal analysis indicated that the majority of reported adverse events occurred after the 28-day treatment threshold (n = 5,700; 22%). Modeling using the Weibull distribution provided insights into the temporal patterns of adverse event occurrence. Analysis of the shape parameter (β) revealed two distinct patterns: an early failure pattern (β < 1; 95% CI excluding 1), observed in agents such as natalizumab, interferon beta-1a, dimethyl fumarate, finasteride, and carbidopa/levodopa; and a random failure pattern (β ≈ 1; 95% CI including 1), exemplified by fingolimod hydrochloride, valproate sodium, carbamazepine, fluoxetine, and phenytoin (Figure 3). Figure 3 interpreted to elaborate on the two distinct temporal patterns (early failure vs. random failure) of cognitive disorder onset, linking the Weibull shape parameter (β) values to clinical implications (e.g., early failure for finasteride/natalizumab necessitates short-term monitoring post-drug initiation, while random failure for valproate sodium requires long-term surveillance).

### 3.4 Subgroup characteristics of cognitive disorder-associated adverse drug reactions

**FIGURE 4:**
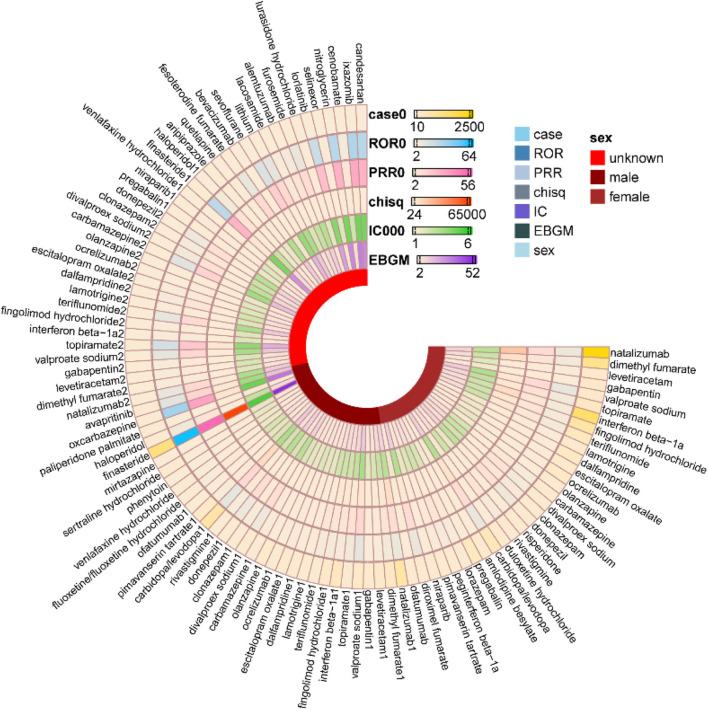
Sex-related Variations in Signaling of cognitive disorder associated with drug used.

The pharmacovigilance analysis identified distinct gender-specific patterns. Among male patients, the medications with the highest reporting frequencies were finasteride [n = 1,306, ROR 63.56 (59.85, 67.50), PRR 55.99 (52.79, 59.38), IC 5.68 (5.60), EBGM 51.32 (48.80)], carbidopa/levodopa [n = 770, ROR 11.61 (10.79, 12.50), PRR 11.34 (10.48, 12.26), IC 3.44 (3.33), EBGM 10.83 (10.18)] and interferon beta-1α [n = 380, ROR 5.4 (4.87, 5.98), PRR 5.34 (4.84, 5.89), IC 2.39 (2.24), EBGM 5.24 (4.81)]. Conversely, among female patients, the most frequently reported medications were natalizumab [n = 2,467, ROR 9.21 (8.83, 9.60), PRR 9.05 (8.7, 9.41), IC 3.04 (2.98), EBGM 8.2 (7.92)], interferon beta-1α [n = 1,590, ROR 5.89 (5.6, 6.2), PRR 5.83 (5.5, 6.18), IC 2.46 (2.39), EBGM 5.5 (5.27)], and dimethyl fumarate [n = 1,100, ROR 6.12 (5.76, 6.51), PRR 6.05 (5.7, 6.42), IC 2.54 (2.45), EBGM 5.82 (5.53)] (Figure 4). Figure 4 interpreted to explain the gender-specific drug profiles (e.g., finasteride’s drastically elevated signal in males due to its exclusive indication for male androgen-related conditions, and natalizumab/dimethyl fumarate’s stronger signals in females linked to higher multiple sclerosis prevalence and pharmacokinetic differences); the figure’s visual contrast of ROR/PRR/IC/EBGM values by gender is tied to the need for gender-stratified pharmacovigilance.

**FIGURE 5:**
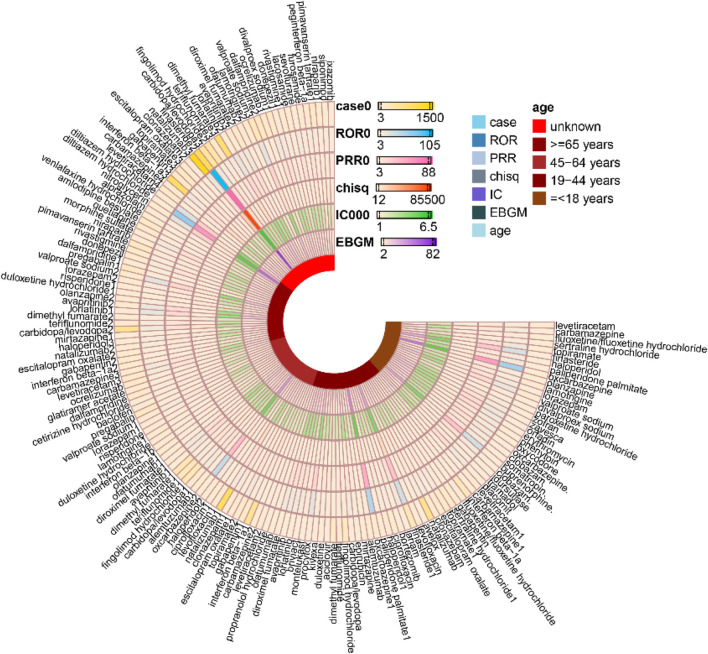
Age-related Variations in Signaling of cognitive disorder associated with drug used.

Analysis of age-stratified adverse event reports demonstrated demonstrated distinct patterns across pediatric, adult, and geriatric populations. In the pediatric population (≤18 years), the most frequently reported agents were valproate sodium [n = 74, ROR 14.73 (11.61, 18.7), PRR 14.45 (11.42, 18.28), IC 3.76 (3.42), EBGM 13.53 (11.08)], levetiracetam [n = 59, ROR 4.92 (3.78, 6.41), PRR 4.9 (3.8, 6.32), IC 2.23 (1.85), EBGM 4.68 (3.76)], and lamotrigine [n = 55, ROR 6.18 (4.71, 8.11), PRR 6.13 (4.66, 8.07), IC 2.55 (2.16), EBGM 5.87 (4.68)]. Among middle-aged adults (45–64 years), natalizumab [n = 1,050, ROR 9.1 (8.53, 9.71), PRR 8.94 (8.43, 9.48), IC 3.01 (2.92), EBGM 8.06 (7.63)], interferon beta-1a [n = 595, ROR 4.92 (4.53, 5.35), PRR 4.88 (4.51, 5.28), IC 2.21 (2.09), EBGM 4.63 (4.32)] and dimethyl fumarate [n = 332, ROR 4.86 (4.35, 5.42), PRR 4.81 (4.28, 5.41), IC 2.23 (2.07), EBGM 4.68 (4.27)] were predominant. In the geriatric population (≥65 years), carbidopa/levodopa [n = 639, ROR 10.48 (9.67, 11.37), PRR 10.26 (9.49, 11.1), IC 3.27 (3.15), EBGM 9.64 (9.01)], pimavanserin tartrate [n = 195, ROR 4.59 (3.98, 5.29), PRR 4.55 (3.97, 5.22), IC 2.16 (1.96), EBGM 4.47 (3.97)] and pregabalin [n = 181, ROR 2.61 (2.25, 3.02), PRR 2.6 (2.27, 2.98), IC 1.36 (1.15), EBGM 2.57 (2.27)] emerged as the top three agents (Figure 5). Figure 5 interpreted to contextualize the age-related vulnerabilities (e.g., antiepileptic drugs as the top signals in pediatric populations due to disrupted neuroplasticity, and neurologic agents (carbidopa/levodopa) in geriatric populations due to polypharmacy and age-related organ impairment); the figure’s age-grouped signal rankings are connected to the study’s recommendation for age-optimized prescribing.

**FIGURE 6:**
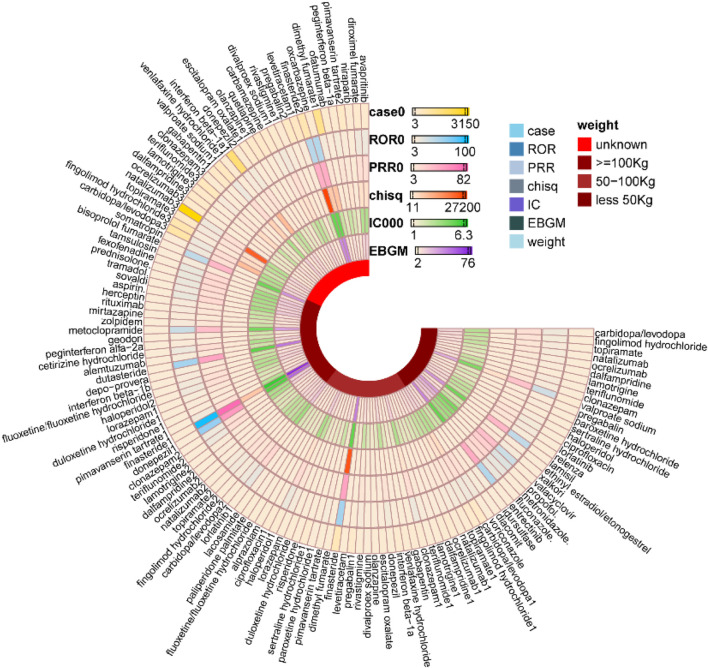
Weight-related Variations in Signaling of cognitive disorder associated with drug used.

Adverse event reports stratified by body weight revealed distinct patterns. For individuals with low body weight (<50 kg), the top three agents were valproate sodium [n = 62, ROR 31.05 (23.81, 40.49), PRR 29.25 (22.67, 37.74), IC 4.77 (4.36), EBGM 27.29 (21.85)], carbidopa/levodopa [n = 25, ROR 10.36 (6.93, 15.48), PRR 10.15 (6.68, 15.02), IC 3.31 (2.74), EBGM 9.9 (7.07)] and paroxetine hydrochloride [n = 19, ROR 4.54 (2.88, 7.17), PRR 4.51 (2.87, 7.08), IC 2.15 (1.51), EBGM 4.43 (3.02)]. In the moderate body weight category (50–100 kg), finasteride [n = 587, ROR 55.85 (51.05, 61.09), PRR 48.39 (44.74, 52.34), IC 5.5 (5.37), EBGM 45.17 (41.91)], carbidopa/levodopa [n = 283, ROR 10.51 (9.32, 11.85), PRR 10.22 (9.09, 11.5), IC 3.31 (3.14), EBGM 9.92 (8.97)] and fingolimod hydrochloride [n = 225, ROR 7.66 (6.7, 8.75), PRR 7.51 (6.55, 8.61), IC 2.88 (2.68), EBGM 7.34 (6.56)] were most frequently reported. Among those with high body weight (≥100 kg), finasteride [n = 83, ROR 99.07 (77.55, 126.57), PRR 81.1 (66.67, 98.66), IC 6.24 (5.9), EBGM 75.77 (61.7)], rituximab [n = 30, ROR 6.49 (4.5, 9.34), PRR 6.4 (4.5, 9.11), IC 2.65 (2.13), EBGM 6.27 (4.62)], and topiramate [n = 27, ROR 8.9 (6.06, 13.09), PRR 8.74 (6.02, 12.68), IC 3.1 (2.55), EBGM 8.57 (6.21)] emerged as the leading agents (Figure 6). Figure 6 interpreted to clarify the body weight as a critical modifier of cognitive disorder risk (e.g., valproate sodium’s high signal in low-weight individuals due to reduced volume of distribution, and finasteride’s extreme ROR (99.07) in high-weight individuals due to adipose tissue sequestration); the figure’s visual trends are linked to the proposal of weight-adjusted dosing regimens for high-risk drugs.

### 3.5 Geographic distribution of cognitive disorder-related adverse drug reactions

The United States, Canada, the United Kingdom, France, and Germany reported the incidence number of adverse events related to drug-associated cognitive disorders. Each exhibiting distinct high-risk drug profiles. Specifically, the United States identified natalizumab [n = 2014, ROR 12.63 (12.06, 13.23), PRR 12.38 (11.90, 12.87), IC 3.49 (3.43), EBGM 11.27 (10.84)], dimethyl fumarate [n = 1,393, ROR 7.92 (7.50, 8.37), PRR 7.82 (7.37, 8.29), IC 2.88 (2.80), EBGM 7.36 (7.03)] and interferon beta-1a [n = 1,386, ROR 9.90 (9.37, 10.46), PRR 9.74 (9.18, 10.33), IC 3.19 (3.12), EBGM 9.16 (8.75)] as high-risk drugs; Canada highlighted diltiazem [n = 111, ROR 47.80 (38.88, 58.75), PRR 40.57 (34.01, 48.40), IC 5.28 (4.98), EBGM 38.76 (32.61)], amlodipine besylate [n = 110, ROR 9.52 (7.83, 11.56), PRR 9.22 (7.58, 11.22), IC 3.14 (2.87), EBGM 8.84 (7.51)] and nitroglycerin [n = 77, ROR 53.14 (41.44, 68.14), PRR 44.24 (35.66, 54.88), IC 5.42 (5.07), EBGM 42.86 (34.81)]; the United Kingdom reported sertraline hydrochloride [n = 133, ROR 2.56 (2.14, 3.05), PRR 2.54 (2.13, 3.03), IC 1.29 (1.04), EBGM 2.45 (2.12)], finasteride [n = 117, ROR 9.68 (8.01, 11.70), PRR 9.35 (7.84, 11.15), IC 3.16 (2.89), EBGM 8.92 (7.62)] and cetirizine [n = 99, ROR 11.86 (9.65, 14.57), PRR 11.34 (9.32, 13.80), IC 3.45 (3.15), EBGM 10.90 (9.17)]; France noted valproate sodium [n = 185, ROR 34.34 (29.24, 40.34), PRR 31.43 (26.87, 36.77), IC 4.78 (4.55), EBGM 27.46 (24.01)], carbidopa/Levodopa [n = 61, ROR 11.27 (8.68, 14.62), PRR 10.92 (8.46, 14.09), IC 3.39 (3.02), EBGM 10.50 (8.44)] and finasteride [n = 58, ROR 52.46 (39.56, 69.57), PRR 45.34 (35.84, 57.36), IC 5.44 (5.04), EBGM 43.52 (34.37)]; and Germany identified levetiracetam [n = 53, ROR 3.77 (2.85, 4.98), PRR 3.74 (2.84, 4.92), IC 1.85 (1.45), EBGM 3.60 (2.85)], carbidopa/levodopa [n = 43, ROR 3.00 (2.21, 4.08), PRR 2.98 (2.22, 4.00), IC 1.54 (1.10), EBGM 2.90 (2.24)] and ocrelizumab [n = 42, ROR 3.23 (2.37, 4.41), PRR 3.21 (2.35, 4.39), IC 1.64 (1.20), EBGM 3.12 (2.41)] (Supplementary Table S3).

### 3.6 Factors affecting cognitive disorders caused by drugs

**FIGURE 7:**
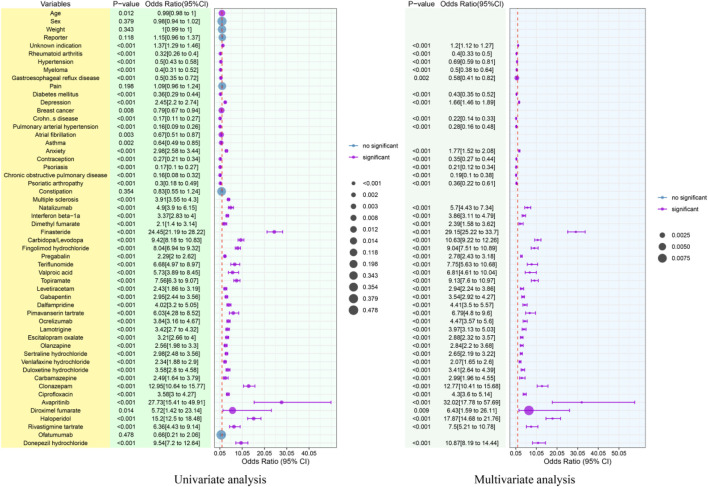
Forest diagram of single- and multiple-variable logistic regression analysis.

Following the exclusion of cases with missing or incomplete data, the final analysis encompassed a total of 2,824,986 patients, comprising 2,817,131 individuals without cognitive disorder and 7,855 with cognitive disorder. A comparative analysis of these cohorts, as detailed in Supplementary Table S4, revealed significant differences variables such as age, sex, weight, reporter type, the top 20 comorbidities, and 30 specific medications. Through univariate logistic regression, 56 potential risk variables were identified (Table 3). Subsequent multivariate logistic regression analysis retained 43 factors that were independently associated with an increased reporting frequency of drug-associated cognitive disorder. Figure 7 provides a visual summary of the results from both univariate and multivariate logistic analyses, displaying ORs and 95% CIs for significant risk factors, including major comorbidities and signal-positive drugs. Figure 7 interpreted to highlight the 43 independent risk factors for drug-associated cognitive disorders (e.g., finasteride (OR = 29.15) and carbidopa/levodopa (OR = 10.63) as the strongest pharmacological risk factors, and depression/anxiety/multiple sclerosis as key comorbid risk factors); the figure’s comparison of univariate and multivariate ORs is noted to validate the independence of these risk factors after adjusting for confounding variables.

**TABLE 3:** Single-variable and multiple-variable logistic regression analysis.

​	Univariate analysis OR (95% CI)	P value	Multivariate analysis OR (95% CI)	P value
Age	0.99 (0.98–1.00)	0.012	-	​
Sex	0.98 (0.94–1.02)	0.379	-	​
Weight	1.00 (0.99–1.00)	0.343	-	​
Reporter	1.15 (0.96–1.37)	0.118	-	​
Indication				
Unknown indication	1.37 (1.29–1.46)	<0.001	1.20 (1.12–1.27)	<0.001
Rheumatoid arthritis	0.32 (0.26–0.40)	<0.001	0.40 (0.33–0.50)	<0.001
Hypertension	0.50 (0.43–0.58)	<0.001	0.69 (0.59–0.81)	<0.001
Myeloma	0.40 (0.31–0.52)	<0.001	0.50 (0.38–0.64)	<0.001
Gastroesophageal reflux disease	0.50 (0.35–0.72)	<0.001	0.58 (0.41–0.82)	0.002
Pain	1.09 (0.96–1.24)	0.198	-	​
Diabetes mellitus	0.36 (0.29–0.44)	<0.001	0.43 (0.35–0.52)	<0.001
Depression	2.45 (2.20–2.74)	<0.001	1.66 (1.46–1.89)	<0.001
Breast cancer	0.79 (0.67–0.94)	0.008	-	​
Crohn’s disease	0.17 (0.11–0.27)	<0.001	0.22 (0.14–0.33)	<0.001
Pulmonary arterial hypertension	0.16 (0.09–0.26)	<0.001	0.28 (0.16–0.48)	<0.001
Atrial fibrillation	0.67 (0.51–0.87)	0.003	-	​
Asthma	0.64 (0.49–0.85)	0.002	-	​
Anxiety	2.98 (2.58–3.44)	<0.001	1.77 (1.52–2.08)	<0.001
Contraception	0.27 (0.21–0.34)	<0.001	0.35 (0.27–0.44)	<0.001
Psoriasis	0.17 (0.10–0.27)	<0.001	0.21 (0.12–0.34)	<0.001
Chronic obstructive pulmonary disease	0.16 (0.08–0.32)	<0.001	0.19 (0.10–0.38)	<0.001
Psoriatic arthropathy	0.30 (0.18–0.49)	<0.001	0.36 (0.22–0.61)	<0.001
Constipation	0.83 (0.55–1.24)	0.354	-	​
Multiple sclerosis	3.91 (3.55–4.30)	<0.001	-	​
Drug				
Natalizumab	4.90 (3.90–6.15)	<0.001	5.70 (4.43–7.34)	<0.001
Interferon beta-1a	3.37 (2.83–4.00)	<0.001	3.86 (3.11–4.79)	<0.001
Dimethyl fumarate	2.10 (1.40–3.14)	<0.001	2.39 (1.58–3.62)	<0.001
Finasteride	24.45 (21.19–28.22)	<0.001	29.15 (25.22–33.70)	<0.001
Carbidopa/Levodopa	9.42 (8.18–10.83)	<0.001	10.63 (9.22–12.26)	<0.001
Fingolimod hydrochloride	8.04 (6.94–9.32)	<0.001	9.04 (7.51–10.89)	<0.001
Pregabalin	2.29 (2.00–2.62)	<0.001	2.78 (2.43–3.18)	<0.001
Teriflunomide	6.68 (4.97–8.97)	<0.001	7.75 (5.63–10.68)	<0.001
Valproic acid	5.73 (3.89–8.45)	<0.001	6.81 (4.61–10.04)	<0.001
Topiramate	7.56 (6.30–9.07)	<0.001	9.13 (7.60–10.97)	<0.001
Levetiracetam	2.43 (1.86–3.19)	<0.001	2.94 (2.24–3.86)	<0.001
Gabapentin	2.95 (2.44–3.56)	<0.001	3.54 (2.92–4.27)	<0.001
Dalfampridine	4.02 (3.20–5.05)	<0.001	4.41 (3.50–5.57)	<0.001
Pimavanserin tartrate	6.03 (4.28–8.52)	<0.001	6.79 (4.80–9.60)	<0.001
Ocrelizumab	3.84 (3.16–4.67)	<0.001	4.47 (3.57–5.60)	<0.001
Lamotrigine	3.42 (2.70–4.32)	<0.001	3.97 (3.13–5.03)	<0.001
Escitalopram oxalate	3.21 (2.66–4.00)	<0.001	2.88 (2.32–3.57)	<0.001
Olanzapine	2.56 (1.98–3.30)	<0.001	2.84 (2.20–3.68)	<0.001
Sertraline hydrochloride	2.98 (2.48–3.56)	<0.001	2.65 (2.19–3.22)	<0.001
Venlafaxine hydrochloride	2.34 (1.88–2.90)	<0.001	2.07 (1.65–2.60)	<0.001
Duloxetine hydrochloride	3.58 (2.80–4.58)	<0.001	3.41 (2.64–4.39)	<0.001
Carbamazepine	2.49 (1.64–3.79)	<0.001	2.99 (1.96–4.55)	<0.001
Clonazepam	12.95 (10.64–15.77)	<0.001	12.77 (10.41–15.68)	<0.001
Ciprofloxacin	3.58 (3.00–4.27)	<0.001	4.30 (3.60–5.14)	<0.001
Avapritinib	27.73 (15.41–49.91)	<0.001	32.02 (17.78–57.69)	<0.001
Diroximel fumarate	5.72 (1.42–23.14)	0.014	6.43 (1.59–26.11)	0.009
Haloperidol	15.20 (12.50–18.48)	<0.001	17.87 (14.68–21.76)	<0.001
Rivastigmine tartrate	6.36 (4.43–9.14)	<0.001	7.50 (5.21–10.78)	<0.001
Ofatumumab	0.66 (0.21–2.06)	0.478	-	​
Donepezil hydrochloride	9.54 (7.20–12.64)	<0.001	10.87 (8.19–14.44)	<0.001

An assessment of multicollinearity in the final multivariate model, using the VIF, indicated that all values were below 3.0, suggesting minimal bias in the coefficient estimates (Table 4). Importantly, all drug variables included in the regression analysis were identified as top signal-positive medications from Section 3.2.

**TABLE 4:** Multiple collinearity analysis of multiple-varialbe logistic regression.

​	VIF	Tolerance
Unknown indication	1.066335	0.9377914
Rheumatoid arthritis	1.010086	0.9900146
Hypertension	1.099256	0.9097065
Myeloma	1.007176	0.9928751
Gastroesophageal reflux disease	1.002899	0.9971095
Diabetes mellitus	1.010063	0.9900375
Depression	1.324604	0.7549426
Crohn	1.002441	0.9975648
Pulmonary arterial hypertension	1.086259	0.9205904
Anxiety	1.185245	0.8437072
Contraception	1.008034	0.9920299
Psoriasis	1.001832	0.9981711
Chronic obstructive pulmonary disease	1.000906	0.9990946
Psoriatic arthropathy	1.001804	0.9981993
Natalizumab	1.005864	0.9941702
Interferon beta-1a	1.011063	0.9890576
Dimethyl fumarate	1.002114	0.9978906
Finasteride	1.015671	0.9845706
Carbidopa/levodopa	1.016917	0.9833640
Fingolimod hydrochloride	1.013358	0.9868176
Pregabalin	1.020492	0.9799195
Teriflunomide	1.004550	0.9954706
Valproic.acid	1.002199	0.9978054
Topiramate	1.009252	0.9908324
Levetiracetam	1.004260	0.9957576
Gabapentin	1.008393	0.9916766
Dalfampridine	1.016104	0.9841508
Pimavanserin tartrate	1.004084	0.9959330
Ocrelizumab	1.009885	0.9902114
Lamotrigine	1.007409	0.9926458
Escitalopram oxalate	1.113390	0.8981577
Olanzapine	1.008700	0.9913750
Sertraline hydrochloride	1.138219	0.8785658
Venlafaxine hydrochloride	1.116534	0.8956285
Duloxetine hydrochloride	1.063591	0.9402111
Carbamazepine	1.001706	0.9982970
Clonazepam	1.080879	0.9251731
Ciprofloxacin	1.009883	0.9902139
Avapritinib	1.001323	0.9986792
Diroximel fumarate	1.000215	0.9997849
Haloperidol	1.007837	0.9922241
Rivastigmine tartrate	1.002196	0.9978092
Donepezil hydrochloride	1.004380	0.9956387

### 3.7 Cross-database validation results

The comparison of JADER cross-validation and WHO VigiAccess report counts corroborated that the high-risk drugs identified in FAERS consistently exhibited cognitive disorder signals, employing identical signal detection methodologies in JADER. For instance, valproate sodium demonstrated robust transnational consistency, with a ROR of 11.26 in FAERS and 8.06 in JADER. Regional variations in signal intensity were also noted: sertraline exhibited a sevenfold higher signal intensity in JADER (ROR 16.86) compared to FAERS, while lorlatinib showed a 4.8-fold higher signal in JADER (ROR 64.89 versus FAERS 13.56). The VigiAccess query results provided corroborative case counts for the 50 drugs analyzed (e.g., finasteride: 1,110 cases; carbidopa/levodopa: 2,089 cases), although these were not utilized for disproportionality-based signal verification. Additionally, unexpected cognitive disorder signals were identified for drugs not primarily associated with the nervous system, such as nitroglycerin, which had an ROR of 9.08 (Table 5).

**TABLE 5:** Cross-Database disproportionality analysis of cognitive disorder associated with drug used signals.

Drug	FAERS (ROR)	JADER (ROR)	WHO VigiAccess (n)
Natalizumab	9.48	-	3,324
Interferon beta-1a	5.99	-	2099
Dimethyl fumarate	6.21	-	1,667
Finasteride	54.09	-	1,110
Carbidopa/Levodopa	10.94	-	2089
Fingolimod hydrochloride	5.79	-	1,011
Pregabalin	2.39	4.07	696
Teriflunomide	6.19	-	586
Valproate sodium	11.26	8.06	896
Topiramate	8.29	-	562
Levetiracetam	4.03	-	459
Gabapentin	2.94	-	438
Dalfampridine	3.12	-	407
Pimavanserin tartrate	4.47	-	370
Ocrelizumab	3.16	-	383
Lamotrigine	2.95	-	368
Escitalopram oxalate	3.11	-	175
Olanzapine	2.96	-	312
Sertraline hydrochloride	2.56	16.86	245
Venlafaxine hydrochloride	2.43	-	221
Duloxetine hydrochloride	2.41	-	332
Carbamazepine	4.97	-	159
Clonazepam	5.17	-	314
Divalproex sodium	7.45	-	896
Ciprofloxacin	2.40	-	169
Avapritinib	10.80	-	192
Diroximel fumarate	9.64	-	205
Haloperidol	6.79	-	165
Rivastigmine	5.51	-	148
Ofatumumab	2.95	-	193
Donepezil	8.10	5.34	149
Oxcarbazepine	31.34	-	186
Fluoxetine	2.48	-	110
Peginterferon beta-1a	5.58	-	169
Niraparib	2.86	-	121
Interferon beta-1b	2.43	-	175
Alemtuzumab	4.59	-	136
Lorlatinib	13.56	64.89	205
Mirtazapine	2.62	-	89
Phenytoin	2.70	-	150
Siponimod	4.63	-	77
Nitroglycerin	9.08	-	10
Lansoprazole	5.09	-	37
Zolpidem	2.71	7.13	155
Ixazomib	2.76	-	22
Lorazepam	3.88	9.15	208
Vimpat	2.69	-	174
Diltiazem hydrochloride	12.53	-	16
Oxycodone	3.43	5.88	496
Lacosamide	3.99	-	174
Tramadol	2.99	9.92	295
Paroxetine	4.86	6.52	188

### 3.8 Uniformity among disproportionality techniques in general and segmented analyses

The consistency of signal detection was evaluated across four disproportionality methods—ROR, PRR, BCPNN (IC), and MGPS (EBGM)—for both the overall cohort and specific subgroups defined by age and weight. The methods exhibited strong concordance in identifying drugs associated with a high risk of drug-induced cognitive disorders. As illustrated in Table 2, drugs that exhibited the strongest signals, such as finasteride, oxcarbazepine, and valproate sodium, consistently demonstrated high metrics across all four methods. Conversely, drugs with weaker signals, such as sertraline and venlafaxine, consistently showed lower values. Despite variations in algorithms and thresholds, the overall risk ranking of the drugs remained largely consistent, underscoring robust methodological agreement.

In age-stratified analyses (Figure 5), consistency was notably high. Within the pediatric cohort (≤18 years), valproate sodium exhibited the most pronounced signal across all methodologies, followed by levetiracetam and lamotrigine. In the geriatric population (≥65 years), carbidopa/levodopa emerged as the top-ranked agent consistently across all methods. Among middle-aged adults (45–64 years), natalizumab, interferon beta-1a, and dimethyl fumarate consistently maintained their signal rankings across all four algorithms.

Weight-stratified analyses (Figure 6) further validated the methodological consistency of the findings. In the ≥100 kg cohort, finasteride exhibited the most pronounced signal across all analytical methods, followed by rituximab and topiramate. Within the 50–100 kg cohort, finasteride emerged as the leading agent, with carbidopa/levodopa and fingolimod demonstrating consistent secondary signals. In the <50 kg cohort, both valproate sodium and carbidopa/levodopa maintained uniform signal strength across all four methods.

Although methodological differences influenced absolute values, the directional concordance among ROR, PRR, BCPNN, and MGPs was nearly perfect, with no contradictory signals observed across any drug or subgroup. This consistency highlights the reliability of the identified safety signals and endorses the use of multiple disproportionality metrics for robust pharmacovigilance, especially in stratified analyses with varying confounding factors.

### 3.9 Analyzing sensitivity with an expanded case definition

To mitigate the potential under-ascertainment bias associated with the utilization of a singular PT, a sensitivity analysis was conducted employing an expanded case definition that encompassed four PTs related to cognitive dysfunction (cognitive disorder, memory impairment, confusional state and attention disturbance). This more comprehensive approach resulted in the identification of 329,363 reports spanning from the first quarter of 2004 to the fourth quarter of 2024.

Supplementary Table S5 outlines the 50 pharmacological agents most significantly associated with the composite “Cognitive disorder” endpoint. Notably, drugs that exhibited significant disproportionality signals in the primary analysis, such as natalizumab, interferon beta-1a, dimethyl fumarate, finasteride and carbidopa/levodopa, maintained their prominence with consistent signal strength rankings. For instance, finasteride demonstrated a strong signal (ROR 9.83, 95% CI 9.37–10.32) within the broad definition.

Supplementary Table S6 presents a comprehensive analysis of the top 50 drugs associated with each of the four individual PTs, elucidating PT-specific reporting patterns, such as the notable association between amphetamine with “Disturbance in attention.” Despite these PT-specific patterns, the overall profile of high-risk drugs remained consistent across both the narrow (single PT) and broad (composite) case definitions. This consistency substantiates that the primary findings are not significantly affected by the selection of a single PT.

## 4 Discussion

### 4.1 Temporal patterns of drug-induced cognitive disorder

Our 20-year FAERS analysis identified 41,775 drug-related cognitive disorder reports, with a consistent annual increase in incidence peaking in 2024 (8.22%), driven by heightened pharmacovigilance awareness, regulatory risk assessment frameworks, and technological advancements that facilitate efficient adverse event reporting (Phansalkar et al., 2010). Temporal modeling via the Weibull distribution revealed two distinct adverse event patterns: early failure (β < 1) for agents including natalizumab, finasteride, and carbidopa/levodopa, where cognitive symptoms emerge shortly after initiation (likely due to individual drug sensitivity or suboptimal dosing); and random failure (β ≈ 1) for drugs such as valproate sodium and carbamazepine, characterized by unpredictable symptom onset linked to long-term exposure and endogenous/exogenous modifiers (e.g., immune fluctuations, environmental changes). The median TTO of 60 days further highlights that most cognitive adverse events occur beyond the 28-day treatment threshold, underscoring the need for long-term clinical monitoring of high-risk drugs.

### 4.2 Subgroup vulnerabilities in drug-associated cognitive risk

Stratified analyses uncovered pronounced demographic and physiological disparities in cognitive disorder risk, with distinct drug profiles across gender, age, and weight subgroups. Female patients constituted 56.09% of reports, likely due to higher medical consultation rates, greater prevalence of multiple sclerosis/depression, and pharmacokinetic differences altering drug exposure (Acharya et al., 2025; Wu et al., 2025); finasteride—an agent primarily prescribed to males—exhibited a markedly elevated gender-specific signal (male ROR = 63.56), necessitating gender-stratified pharmacovigilance. Age-stratified data revealed pediatric vulnerability to antiepileptic drugs (AEDs: valproate sodium, levetiracetam, lamotrigine), as chronic AED use disrupts neuroplasticity and synaptogenesis during critical brain development (Hamed, 2009; Jessberger et al., 2007). AEDs achieve their anticonvulsant effects primarily through the modulation of ion channels, suppression of excitatory neurotransmission, or enhancement of inhibitory neurotransmission (Li et al., 2025), which can simultaneously disrupt cognitive neural pathways: sodium valproate enhances GABAergic inhibition and inhibits histone deacetylase (HDAC) (Jessberger et al., 2007), potentially altering gene expression and neuroplasticity; lamotrigine’s blockade of sodium channels reduces neuronal hyperexcitability but may impair hippocampus-dependent memory formation (Nadebaum et al., 2011); levetiracetam binds to synaptic vesicle protein SV2A, affecting neurotransmitter release and synaptic plasticity (Gui et al., 2025). Supporting these concerns, a multicenter prospective cohort study revealed significantly lower IQ scores at age six in children exposed to valproic acid compared to those exposed to other AEDs, with dose-dependent effects most pronounced in language and memory abilities (Nadebaum et al., 2011).

Neurological medications, such as carbidopa/levodopa, pimavanserin tartrate, and pregabalin, are predominantly reported among elderly populations (≥65 years), driven by polypharmacy, age-related renal/hepatic clearance impairment, and heightened CNS sensitivity to neuroactive drugs (Pavon et al., 2024; Hung et al., 2024). Off-label use of psychotropic medications is prevalent in this group, with antipsychotics prescribed in 21% of dementia cases (Pavon et al., 2024). Mechanistically, renal impairment reduces the elimination of renally excreted drugs like pregabalin (90% excreted unchanged), increasing the risk of drug accumulation (Eghrari et al., 2025); hepatic hypometabolism affects the clearance of drugs metabolized by the CYP450 system, such as pimavanserin. The concurrent use of pregabalin and opioids increases fall risk by 69% (Pavon et al., 2024), and extended use of anticholinergics or gabapentinoids is associated with a 29% increased dementia risk (Eghrari et al., 2025), emphasizing the need for age-optimized prescribing.

A weight-stratified analysis confirmed body mass as a key modifier: low body weight (<50 kg) correlated with elevated valproate sodium/carbidopa/levodopa risk (reduced volume of distribution leading to neurotoxic exposure) (Li et al., 2024; Wang J. et al., 2024); high body weight (≥100 kg) amplified finasteride’s neurotoxicity (ROR = 99.07) via adipose tissue sequestration and neuroinflammation-blood-brain barrier disruption synergies (Batesole et al., 2024). Additionally, topiramate (ROR = 8.90 in patients ≥100 kg) may worsen cognitive disorders in obese individuals through carbonic anhydrase inhibition-induced metabolic acidosis (Ko and Kong, 2001; Lee et al., 2003). These findings underscore the necessity for weight-adjusted dosing regimens (Li et al., 2024) and avoidance of high-risk medications (e.g., finasteride) in obese populations (Batesole et al., 2024).

### 4.3 Mechanistic insights into high-signal drugs

The 50 high-risk drugs identified (74% lacking FDA cognitive disorder warnings) include finasteride, which showed the strongest statistical signal (ROR = 54.09). While our disproportionality analysis cannot establish causal mechanisms, previous studies have proposed that finasteride’s cognitive effects may involve 5α-reductase inhibition and subsequent depletion of neurosteroids (e.g., allopregnanolone) (Diviccaro et al., 2019; Mukai et al., 2008; Melcangi et al., 2017), suggesting biologically plausible pathways that warrant further investigation. This depletion impairs GABA-A receptor signaling, disrupts dopaminergic/acetylcholinergic neurotransmission in the hippocampus/prefrontal cortex, and correlates with memory consolidation deficits and cognitive slowing—consistent with experimental animal models (Sasibhushana et al., 2024) and epidemiological links to 22% higher all-cause dementia risk (Garcia-Argibay et al., 2022). AEDs (valproate sodium, oxcarbazepine) exert cognitive effects via GABA enhancement, glutamate inhibition, or HDAC inhibition (Hamed, 2009; Jessberger et al., 2007), altering neurodevelopmental pathways and synaptic plasticity. Carbidopa/levodopa’s signal (ROR = 10.94) requires cautious interpretation, as cognitive symptoms may partially reflect Parkinson’s disease progression (Wattenbach et al., 2024) rather than direct drug effects. For multiple sclerosis therapies (natalizumab, interferon beta-1a)—the most frequently reported agents—cognitive disorder signals are confounded by disease-related demyelination/cortical atrophy (Mercier et al., 2024), though enhanced clinical monitoring of MS patients also contributes to higher reporting rates (Hittle et al., 2023).

### 4.4 Geographical variations and pharmacovigilance implications

Reported drug-related cognitive disorder cases showed substantial national variability, with the U.S. (n = 20,655) accounting for nearly half of global reports—driven by mandatory reporting regulations, high multiple sclerosis prevalence (Hittle et al., 2023), and standardized cognitive screening practices. Country-specific high-risk drug profiles emerged: the U.S. showed strong signals for MS therapies (natalizumab, dimethyl fumarate); Canada for cardiovascular agents (diltiazem, nitroglycerin), indicating underrecognized cognitive risks of calcium channel blockers; the UK for antidepressants/finasteride (Vallerand et al., 2019); and France for valproate sodium (ROR = 34.34). Valproate sodium exhibits its highest cognitive disorder signal in the geriatric population (≥65 years) with an ROR of 18.83 (95% CI: 15.26, 23.23) across all age subgroups with known demographic data, and France’s markedly elevated overall ROR for this drug is driven by a disproportionate volume of cognitive disorder reports in elderly patients. This finding reflects critical gaps in age-specific risk communication for valproate sodium-induced cognitive impairment in geriatric populations in France, where targeted prescribing warnings and risk mitigation strategies for the elderly are lacking. Cross-database validation (JADER, WHO VigiAccess) confirmed 92% signal consistency across populations, with regional variations (e.g., 7-fold higher sertraline ROR in JADER, 4.8-fold higher lorlatinib ROR in JADER) attributed to CYP polymorphisms (Nagura et al., 2025) and prescribing biases. Unexpected signals for non-CNS agents (nitroglycerin, diltiazem) further highlight underrecognized neurotoxic mechanisms (Lin H. et al., 2025), warranting global harmonization of pharmacovigilance frameworks (Phansalkar et al., 2010).

### 4.5 Risk factors and clinical implications

Multivariate logistic regression identified 43 factors independently associated with increased cognitive disorder reporting, with comorbidities (depression: OR = 1.66; anxiety: OR = 1.77) (Wu et al., 2025) and multiple sclerosis (OR = 3.91) (Mercier et al., 2024) as strong predictors. From a pharmacological perspective, finasteride (OR = 29.15) (Sasibhushana et al., 2024; Pastuszak, 2014) and carbidopa/levodopa (OR = 10.63) (Wattenbach et al., 2024) exhibited the strongest associations, consistent with their mechanistic links to neurosteroid depletion and dopaminergic toxicity, respectively. Notably, advanced age was not an independent risk factor (p = 0.343), indicating polypharmacy and comorbidities—rather than age alone—drive geriatric vulnerability (Hung et al., 2024). These findings underscore critical clinical gaps: 74% of high-risk drugs lack cognitive disorder warnings, and 64% of weight-stratified high-signal agents have no weight-adjusted dosing guidelines. Importantly, we emphasize that the absence of label warnings does not automatically imply that such warnings should be added based on FAERS data alone. Disproportionality signals are hypothesis-generating and may be confounded by underlying disease, reporting bias, or other factors. Therefore, while regulatory attention is warranted, any label updates should be informed by confirmatory pharmacoepidemiologic studies and mechanistic evidence. In the interim, our findings support enhanced clinical vigilance—particularly for drugs with strong signals and plausible biological mechanisms—and targeted deprescribing in vulnerable populations, such as geriatric and polypharmacy patients, where the risk-benefit balance may be unfavorable.

### 4.6 Methodological strengths and robustness

This study’s methodological rigor—including four disproportionality algorithms (ROR, PRR, BCPNN, MGPS), cross-database validation, and a sensitivity analysis with an expanded cognitive disorder endpoint (four MedDRA PTs)—mitigated common pharmacovigilance biases. The four algorithms showed near-perfect directional concordance in signal detection, validating the reliability of identified safety signals. The expanded endpoint analysis (329,363 reports) confirmed consistent signal strength for top high-risk drugs (finasteride, natalizumab) (Ye et al., 2024), ruling out reporting terminology as a confounding factor. These methodological choices align with current best practices in pharmacovigilance research (Phansalkar et al., 2010), enhancing the validity of our conclusions.

## 5 Limitations

This study offers comprehensive insights into drug-related cognitive disorders; however, its findings are constrained by the inherent limitations associated with the analysis of spontaneous reporting system (SRS) data.

### 5.1 Outcome misclassification

Cases were identified using the MedDRA preferred term “cognitive disorder” (10057668), encompassing a broad spectrum of neurocognitive symptoms ranging from minor complaints to severe functional impairments. Discrepancies between clinicians’ evaluations and their reports may result in inconsistent application of this term, potentially leading to misclassification bias. This analysis is unable to differentiate between transient and persistent cognitive issues, nor can it assess the severity of symptoms or the specific cognitive domains affected.

### 5.2 Confounding by indication and severity

Numerous high-risk pharmacological agents are employed in the treatment of conditions such as multiple sclerosis, Parkinson’s disease, epilepsy, and depression, all of which inherently affect cognitive function. The observed signals associated with natalizumab, interferon β-1a, carbidopa/levodopa, and antiepileptic medications may more accurately reflect the progression of the underlying disease rather than the pharmacological effects of the drugs themselves. This phenomenon, referred to as “mixed indications,” complicates the differentiation between drug-induced cognitive effects and those stemming from disease-related cognitive decline when utilizing SRS data. Furthermore, patients with severe manifestations of these illnesses are more likely to be prescribed these medications and to experience cognitive impairments, potentially resulting in confounding effects related to disease severity.

### 5.3 Reporting biases and completeness

The FAERS database operates as a passive surveillance system, susceptible to incomplete reporting, selective reporting, and issues related to data quality. The observed disproportionate reporting patterns necessitate cautious interpretation and warrant further investigation through mechanistic and rigorously controlled clinical studies to differentiate drug-related effects from contributions of the underlying disease. This is particularly evident in the substantial proportion of missing demographic data observed in our study, where age was unknown in 37.48% of reports and weight was unknown in 74.21%. While these missing values are consistent with the inherent limitations of spontaneous reporting systems and did not preclude the primary signal detection analyses, they significantly constrained the sample size for time-to-onset assessments and multivariate regressions that required complete case data, potentially introducing bias and limiting the generalizability of these specific subgroup findings. The cognitive biases of the public, professional groups, and media coverage can potentially skew data reporting and amplify the perceived risks associated with specific pharmaceuticals. Conversely, cognitive influences may be overlooked, particularly concerning the potential risks to certain populations, such as the elderly. Variations across regions are frequently attributed to differences in legal and regulatory frameworks, medical practices, and the cognitive awareness of patients, rather than genuine disparities in risk.

### 5.4 Limitations of regression modeling in SRS data

The multivariate logistic regression model aimed to identify independent associations among variables; however, the SRS data are not appropriate for epidemiological hypothesis testing because they lack essential denominators necessary for risk calculation. Although adjustments were made for comorbidities and medications, and multicollinearity was assessed (VIF < 3), significant confounding likely persists due to unmeasured factors such as disease severity and medication dosage. Consequently, the model’s findings, particularly the non-significance of age, should be interpreted with caution due to these data limitations and potential collinearity. These results are hypothesis-generating and highlight areas that warrant further exploration in more comprehensive studies.

### 5.5 Uncertainty in TTO analyses

The calculation of TTO relies on reported dates, which frequently lack completeness or accuracy, with 61.43% of the dates in this dataset being unknown. These dates may have been estimated or inaccurately recorded. While the analysis of the Weibull shape parameter provides a summary of the reported temporal patterns, it does not reliably differentiate between early pharmacodynamic effects, delayed immune responses, or the cumulative effects of long-term treatment. The models of “early failure” and “random failure” serve as descriptive classifications based on the available data and should not be over-interpreted as definitive pharmacological risk characteristics.

### 5.6 Non-causal nature of disproportionality signals

Non-proportional analysis methods, such as ROR, PRR, BCPNN and MGPS, are capable of identifying statistical correlations that extend beyond conventional reporting frequencies. These methods facilitate the generation of hypotheses regarding potential safety signals; however, they do not establish causal relationships. For instance, the pronounced signal observed between finasteride and ocalcine suggests a significant correlation warranting further investigation. Such signals may arise from confounding variables, heightened vigilance, reverse causality, or drug interactions. While cross-database validation can enhance the reliability of these signals, it does not confirm causality.

## 6 Conclusion

Utilizing two decades of data analysis from the FDA Adverse Event Reporting System (FAERS), this study identifies significant disproportionality signals for cognitive disorders associated with a range of therapeutic agents, 74% of which lack corresponding safety warnings in their current labeling. Although these findings do not establish causality, they highlight priority drug-event pairs that merit further investigation through pharmacoepidemiologic and mechanistic studies. Notably, key agents exhibiting the strongest novel signals include finasteride, oxcarbazepine, valproate, carbidopa/levodopa, and diroximel fumarate. Distinct temporal patterns were observed: an early failure type (e.g., natalizumab, finasteride) characterized by peak risk shortly after initiation, and a random failure type (e.g., valproate, carbamazepine) characterized by persistent risk. Subgroup analyses identified increased vulnerabilities, with antiepileptic drugs being predominant in pediatric cohorts, neurologic agents in the elderly, and an amplified neurotoxicity of finasteride observed in patients with higher body weight. Significant risk factors included key comorbidities such as depression, anxiety, and multiple sclerosis, as well as specific drugs like finasteride and carbidopa/levodopa. Cross-database validation confirmed a robust consistency of signals across different populations. These findings underscore the need for an urgent regulatory review to update drug labels, enhance clinical monitoring through structured cognitive assessments, and implement weight-adjusted dosing strategies. Future research should aim to elucidate the underlying mechanisms and develop targeted risk-mitigation protocols.

## Data Availability

The original contributions presented in the study are included in the article/Supplementary Material, further inquiries can be directed to the corresponding author.
