# Supplementary material for: Spectrum and signals of medication-associated cognitive disorder: a comprehensive disproportionality analysis with cross-database validation
Source: Front Pharmacol. 2026 Apr 10;17:1762761. doi: 10.3389/fphar.2026.1762761 (PMC13106381; doi:10.3389/fphar.2026.1762761)
Supplement: Supplementary file 1 [file Table1.docx]

**Table S1** Signal Detection 2x2 Table

|  | Drug-related AEs | Non-drug-related AEs | Total |
| --- | --- | --- | --- |
| Drug | $a$ | $b$ | $a+b$ |
| Non-drug | $c$ | $d$ | $c+d$ |
| Total | $a+c$ | $b+d$ | $N=a+b+c+d$ |

Note:

a: Count of adverse events related to drug in the medication group.

b: Count of non-drug-related adverse events in the medication group.

a+b: The drug group had a total count of AEs.

c: Count of adverse events related to drugs in the group not receiving medication.

d: Count of adverse events not related to drugs in the non-drug group.

c+d: Total adverse events recorded in the group without the drug.

a+c: The overall count of drug-related adverse events in all groups.

b+d: The overall count of non-drug-related adverse events in all groups.

N: The overall count of all study participants (sum of a, b, c, d).
