# Supplementary figures and images for: Spectrum and signals of medication-associated cognitive disorder: a comprehensive disproportionality analysis with cross-database validation
Source: Front Pharmacol. 2026 Apr 10;17:1762761. doi: 10.3389/fphar.2026.1762761 (PMC13106381; doi:10.3389/fphar.2026.1762761)

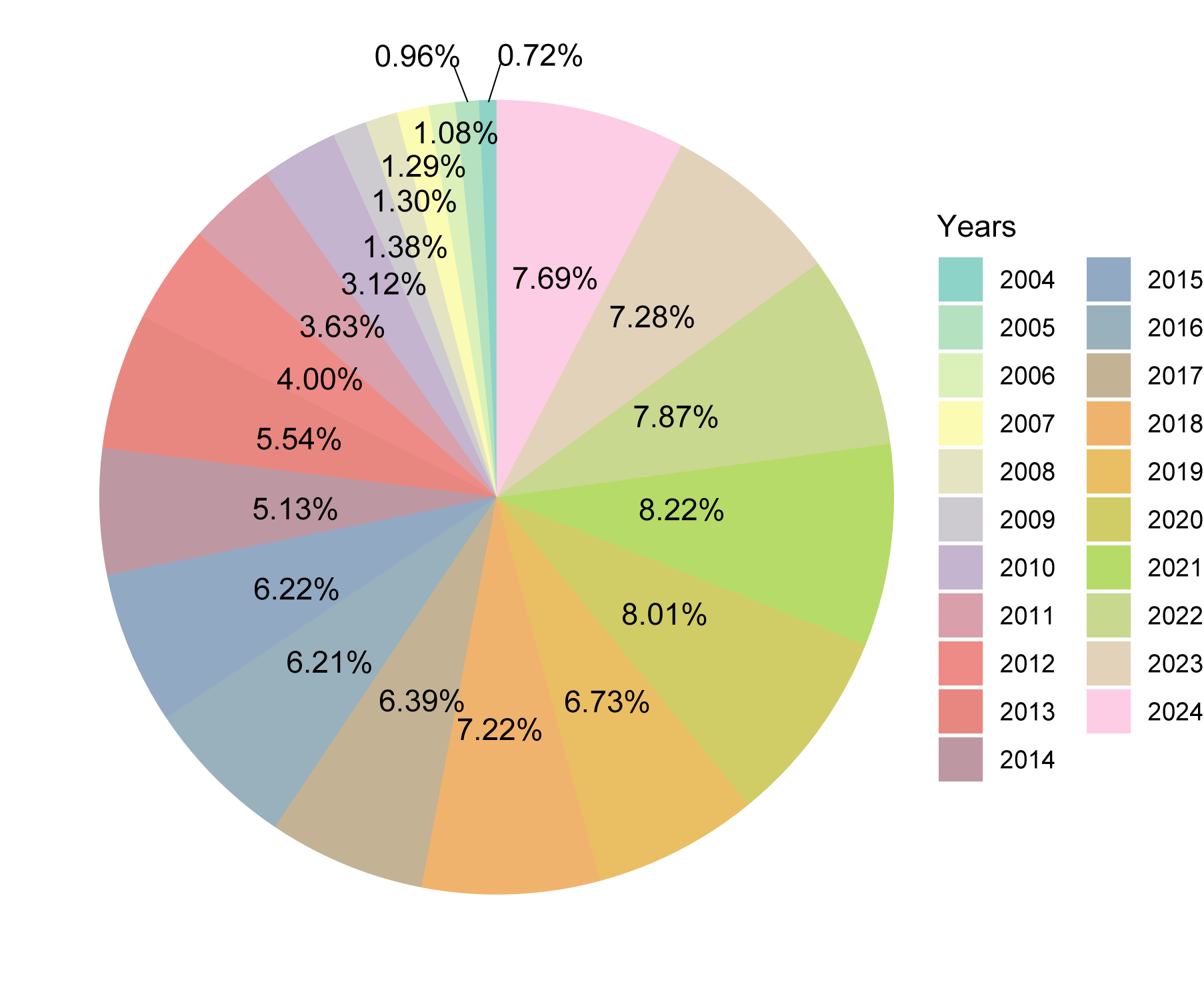

Supplement: Supplementary file 2 [file Image1.tif]
