# Supplementary material for: Spectrum and signals of medication-associated cognitive disorder: a comprehensive disproportionality analysis with cross-database validation
Source: Front Pharmacol. 2026 Apr 10;17:1762761. doi: 10.3389/fphar.2026.1762761 (PMC13106381; doi:10.3389/fphar.2026.1762761)
Supplement: Supplementary file 3 [file Table2.docx]

**Table S2** Comparative Signal Detection in Pharmacovigilance: A Multi-Algorithm Approach Using ROR, PRR, IC and EBGM

| Algorithms | Calculation formula | Criteria |
| --- | --- | --- |
| ROR | $ROR=\frac{a/c}{b/d}=\frac{ad}{bc}$  $95\%CI=e^{In(ROR)\pm1.96\sqrt{\frac{1}{a}+\frac{1}{b}+\frac{1}{c}+\frac{1}{d}}}$ | $a\geq3$  $ROR\geq2$  $95\%CI>1$ |
| PRR | $PRR=\frac{a/(a+b)}{c/(c+d)}=\frac{a(c+d)}{c(a+b)}$  $\chi^{2}=\frac{\left( \left\vert ad-bc \right\vert-\frac{n}{2} \right)^{2}n}{(a+b)(a+c)(c+d)(b+d)}$  $n=a+b+c+d$ | $a\geq3$  $PRR\geq2$  $\chi^{2}\geq4$ |
| BCPNN | $E\left( IC \right)={log}_{2}\frac{\left( C_{xy}+\gamma_{11} \right)\left( C+\alpha\right)\left( C+\beta\right)}{\left( C+\gamma\right)\left( C_{x}+\alpha_{1} \right)\left( C_{y}+\beta_{1} \right)}$  $V\left( IC \right)=\frac{1}{{(In2)}^{2}}\left\{ \left[ \frac{C-C_{xy}+\gamma-\gamma_{11}}{\left( C_{xy}+\gamma_{11} \right)\left( 1+C+\gamma\right)} \right]+\left[ \frac{C-C_{x}+\alpha-\alpha_{1}}{\left( C_{x}+\alpha_{1} \right)\left( 1+C+\alpha\right)} \right]+\left[ \frac{C-C_{x}+\alpha-\alpha_{1}}{\left( C_{y}+\beta_{1} \right)\left( 1+C+\beta\right)} \right] \right\}$  $\gamma=\gamma_{11}\frac{(C+\alpha)(C+\beta)}{(C_{x}+\alpha_{1})(C_{y}+\beta_{1})}$  $IC-2SD=E\left( IC \right)-2\sqrt{V(IC)}$  $\alpha_{1}=\beta_{1}=1$  $\alpha=\beta=2$  $\gamma_{11}=1$  $C=a+b+c+d$  $C_{x}=a+b$  $C_{y}=a+c$  $C_{xy}=a$ | $a\geq3$  $IC-2SD>0$ |
| EBGM^*^ | $EBGM=a(a+b+c+d)/[(a+c)(a+b)]$ | $a>0$  $95\%CI>2$ |

*MGPS/EBGM employs Bayesian methods to compute an empirical Bayes geometric mean (EBGM) estimate of the relative reporting strength for a specific drug-adverse event pair and its uncertainty. This estimate integrates the overall reporting pattern within the database as a dynamic prior (baseline) with the observed report count (likelihood). The method is particularly effective for analyzing sparse data from rare events, utilizing contraction estimation to adjust estimates toward the overall mean.

*IC: Information Component

Note:

ROR: Reporting Odds Ratio, which indicates the strength of the link between a medication and an adverse event.

95%CI: Confidence Interval, indicating the trustworthiness of the estimate.

PRR: Proportional Reporting Ratio, a measure of the strength of the link between a drug and an adverse event.

χ^2^: The chi-square statistic is employed to evaluate the statistical significance of a relationship.

n: Overall count of all research participants (equivalent to N in Table S1)

C: Overall count of all participants in the study (equivalent to n and N).

a: The count of adverse events related to drugs in the drug group (as shown in Table S1).

ROR≥2: A potential signal is defined by an ROR value of 2 or higher.

95%CI>1: The threshold for identifying a potential signal is when the 95% CI lower limit is greater than 1.

PRR≥2: A potential signal is defined by a PRR value of 2 or higher.

χ^2^≥4: A potential signal is defined by a chi-square statistic of 4 or higher, which indicates statistical significance.

E (IC): The Expected Value of Information Component (IC) is a measure used to quantify the strength of the association between a drug and an adverse event.

V (IC): Variance of IC.

γ: Parameters derived from Bayesian priors.

IC-2SD: IC less two standard deviations, a benchmark for identifying signals.

α_1_, β_1_: Bayesian prior parameters set to fixed values (both equal to 1).

α, β: Bayesian prior parameters are set to a fixed value of 2.

γ_11_: Constant Bayesian prior parameter set to 1.

C_x_: The total count of adverse events in the medication group (equivalent to a+b in Table S1).

C_y_: The overall count of drug-related adverse events in all groups (equivalent to a+c in Table S1).

C_xy_: The count of drug-related adverse events in the drug group (identical to a).

IC-2SD>0: A potential signal is defined when IC minus 2SD is greater than zero.

EBGM: Empirical Bayes Geometric Mean, an estimate of association strength tailored for infrequent or rare event data.

a>0: A potential signal is defined by having at least one reported instance of the drug-adverse event pair.

95%CI>2: A potential signal is defined when the lower bound of the 95% confidence interval exceeds 2.
