# Supplementary material for: Spectrum and signals of medication-associated cognitive disorder: a comprehensive disproportionality analysis with cross-database validation
Source: Front Pharmacol. 2026 Apr 10;17:1762761. doi: 10.3389/fphar.2026.1762761 (PMC13106381; doi:10.3389/fphar.2026.1762761)
Supplement: Supplementary file 4 [file Table5.docx]

**Table S5** The primary 50 drugs linked to drug induced-“cognitive disorder” (include cognitive disorder, memory impairment, confusional state and disturbance in attention)

| **Drug** | **Case Reports** | **ROR (95% CI)** | **PRR (95% CI)** | **IC (IC025)** | **EBGM (EBGM05)** |
| --- | --- | --- | --- | --- | --- |
| Natalizumab | 12036 | 4.43 (4.35, 4.51) | 4.18 (4.1, 4.26) | 2.02 (2.00) | 4.07 (4.00) |
| Interferon beta-1a | 9536 | 3.71 (3.63, 3.79) | 3.54 (3.47, 3.61) | 1.80 (1.76) | 3.47 (3.41) |
| Dimethyl fumarate | 7285 | 4.10 (4.00, 4.20) | 3.89 (3.81, 3.97) | 1.94 (1.90) | 3.83 (3.75) |
| Pregabalin | 6626 | 3.42 (3.34, 3.51) | 3.28 (3.22, 3.34) | 1.70 (1.66) | 3.24 (3.17) |
| Fingolimod | 4556 | 3.41 (3.31, 3.51) | 3.27 (3.21, 3.33) | 1.70 (1.65) | 3.24 (3.16) |
| Duloxetine | 4462 | 5.90 (5.72, 6.09) | 5.44 (5.33, 5.55) | 2.43 (2.38) | 5.38 (5.24) |
| Pimavanserin | 4204 | 7.03 (6.81, 7.26) | 6.36 (6.24, 6.49) | 2.65 (2.61) | 6.29 (6.12) |
| Carbidopa/Levodopa | 3506 | 3.79 (3.66, 3.92) | 3.61 (3.47, 3.75) | 1.84 (1.79) | 3.58 (3.48) |
| Gabapentin | 3431 | 3.04 (2.93, 3.14) | 2.93 (2.82, 3.05) | 1.54 (1.49) | 2.91 (2.83) |
| Quetiapine | 3346 | 2.52 (2.43, 2.61) | 2.45 (2.36, 2.55) | 1.29 (1.24) | 2.44 (2.37) |
| Paroxetine | 2846 | 4.40 (4.23, 4.57) | 4.15 (3.99, 4.32) | 2.04 (1.99) | 4.12 (3.99) |
| Olanzapine | 2747 | 3.28 (3.16, 3.41) | 3.16 (3.04, 3.29) | 1.65 (1.59) | 3.14 (3.04) |
| Sodium oxybate | 2700 | 2.27 (2.19, 2.36) | 2.22 (2.13, 2.31) | 1.15 (1.09) | 2.21 (2.14) |
| Venlafaxine | 2684 | 3.23 (3.11, 3.36) | 3.11 (2.99, 3.23) | 1.63 (1.57) | 3.09 (3.00) |
| Dalfampridine | 2568 | 2.55 (2.45, 2.66) | 2.49 (2.39, 2.59) | 1.31 (1.25) | 2.47 (2.39) |
| Sertraline | 2539 | 2.88 (2.76, 2.99) | 2.79 (2.68, 2.90) | 1.47 (1.41) | 2.77 (2.68) |
| Levothyroxine sodium | 2137 | 2.77 (2.65, 2.89) | 2.69 (2.59, 2.80) | 1.42 (1.36) | 2.67 (2.58) |
| Teriflunomide | 2089 | 3.15 (3.01, 3.29) | 3.03 (2.91, 3.15) | 1.6 (1.53) | 3.02 (2.91) |
| Levetiracetam | 2073 | 2.27 (2.17, 2.38) | 2.22 (2.13, 2.31) | 1.15 (1.08) | 2.22 (2.14) |
| Lamotrigine | 2043 | 2.28 (2.18, 2.38) | 2.23 (2.14, 2.32) | 1.15 (1.09) | 2.22 (2.14) |
| Topiramate | 2021 | 4.52 (4.32, 4.73) | 4.26 (4.10, 4.43) | 2.08 (2.02) | 4.24 (4.08) |
| Finasteride | 1948 | 9.83 (9.37, 10.32) | 8.51 (8.18, 8.85) | 3.08 (3.01) | 8.47 (8.13) |
| Tramadol | 1929 | 2.92 (2.79, 3.06) | 2.83 (2.72, 2.94) | 1.49 (1.43) | 2.82 (2.71) |
| Ciprofloxacin | 1846 | 2.78 (2.65, 2.91) | 2.69 (2.59, 2.80) | 1.42 (1.36) | 2.68 (2.58) |
| Amphetamine | 1787 | 7.23 (6.88, 7.60) | 6.52 (6.27, 6.78) | 2.70 (2.63) | 6.49 (6.22) |
| Zolpidem | 1469 | 3.98 (3.77, 4.20) | 3.78 (3.56, 4.01) | 1.91 (1.84) | 3.77 (3.60) |
| Citalopram | 1408 | 3.31 (3.13, 3.49) | 3.18 (3.00, 3.37) | 1.66 (1.59) | 3.17 (3.03) |
| Alprazolam | 1348 | 2.42 (2.29, 2.56) | 2.36 (2.23, 2.50) | 1.24 (1.16) | 2.36 (2.25) |
| Methylphenidate | 1199 | 3.29 (3.11, 3.49) | 3.17 (2.99, 3.36) | 1.66 (1.58) | 3.16 (3.01) |
| Escitalopram | 1185 | 3.13 (2.95, 3.32) | 3.02 (2.85, 3.20) | 1.59 (1.51) | 3.01 (2.87) |
| Rivastigmine | 1171 | 5.47 (5.15, 5.81) | 5.07 (4.78, 5.38) | 2.34 (2.25) | 5.06 (4.81) |
| Lisdexamfetamine Dimesylate | 1164 | 3.93 (3.71, 4.18) | 3.74 (3.53, 3.97) | 1.90 (1.81) | 3.73 (3.55) |
| Fluoxetine | 1154 | 2.64 (2.49, 2.80) | 2.57 (2.42, 2.73) | 1.36 (1.27) | 2.56 (2.44) |
| Mirtazapine | 1145 | 3.64 (3.43, 3.87) | 3.48 (3.28, 3.69) | 1.80 (1.71) | 3.47 (3.30) |
| Valproic acid | 1144 | 4.60 (4.33, 4.89) | 4.33 (4.08, 4.59) | 2.11 (2.02) | 4.32 (4.10) |
| Montelukast | 1082 | 2.83 (2.66, 3.01) | 2.74 (2.58, 2.91) | 1.45 (1.36) | 2.73 (2.60) |
| Memantine | 1027 | 7.91 (7.41, 8.44) | 7.05 (6.65, 7.48) | 2.81 (2.72) | 7.03 (6.66) |
| Carbamazepine | 1020 | 2.79 (2.62, 2.98) | 2.71 (2.56, 2.87) | 1.44 (1.34) | 2.70 (2.57) |
| Atomoxetine | 934 | 3.16 (2.96, 3.38) | 3.04 (2.87, 3.22) | 1.60 (1.51) | 3.04 (2.88) |
| Diroximel | 702 | 4.82 (4.46, 5.21) | 4.52 (4.18, 4.89) | 2.17 (2.06) | 4.51 (4.23) |
| Divalproex sodium | 693 | 3.17 (2.94, 3.42) | 3.05 (2.82, 3.30) | 1.61 (1.50) | 3.05 (2.86) |
| Avapritinib | 661 | 4.92 (4.54, 5.33) | 4.60 (4.25, 4.98) | 2.20 (2.09) | 4.60 (4.30) |
| Rifaximin | 655 | 2.97 (2.74, 3.21) | 2.87 (2.65, 3.10) | 1.52 (1.41) | 2.86 (2.68) |
| Lacosamide | 648 | 2.56 (2.37, 2.78) | 2.50 (2.31, 2.70) | 1.32 (1.20) | 2.49 (2.33) |
| Digoxin | 590 | 3.36 (3.09, 3.65) | 3.22 (2.98, 3.48) | 1.69 (1.57) | 3.22 (3.00) |
| Vortioxetine | 479 | 3.67 (3.35, 4.03) | 3.51 (3.25, 3.80) | 1.81 (1.67) | 3.50 (3.24) |
| Siponimod | 438 | 3.27 (2.97, 3.60) | 3.15 (2.86, 3.47) | 1.65 (1.51) | 3.14 (2.90) |
| Interferon alfa-2b | 412 | 4.04 (3.66, 4.47) | 3.84 (3.48, 4.24) | 1.94 (1.79) | 3.83 (3.52) |
| Axicabtagene ciloleucel | 345 | 3.03 (2.72, 3.38) | 2.93 (2.66, 3.23) | 1.55 (1.39) | 2.93 (2.67) |
| Ziconotide | 286 | 8.46 (7.47, 9.58) | 7.48 (6.65, 8.41) | 2.90 (2.72) | 7.47 (6.74) |

“
