# Supplementary material for: Spectrum and signals of medication-associated cognitive disorder: a comprehensive disproportionality analysis with cross-database validation
Source: Front Pharmacol. 2026 Apr 10;17:1762761. doi: 10.3389/fphar.2026.1762761 (PMC13106381; doi:10.3389/fphar.2026.1762761)
Supplement: Supplementary file 5 [file Table3.docx]

**Table S3** Prevalence of Drug Use in Leading 5 Countries Reporting Drug-Induced Cognitive Disorder

| Drug | Case Reports | ROR (95% CI) | PRR (95% CI) | IC (IC025) | EBGM (EBGM05) | Country |
| --- | --- | --- | --- | --- | --- | --- |
| Natalizumab | 2014 | 12.63(12.06, 13.23) | 12.38(11.90, 12.87) | 3.49(3.43) | 11.27(10.84) | United States |
| Dimethyl fumarate | 1393 | 7.92(7.50, 8.37) | 7.82(7.37, 8.29) | 2.88(2.80) | 7.36(7.03) | United States |
| Interferon beta-1a | 1386 | 9.90(9.37, 10.46) | 9.74(9.18, 10.33) | 3.19(3.12) | 9.16(8.75) | United States |
| Finasteride | 954 | 112.85(105.10, 121.17) | 93.62(88.27, 99.29) | 6.48(6.38) | 89.35(84.18) | United States |
| Teriflunomide | 518 | 7.82(7.16, 8.54) | 7.72(7.14, 8.35) | 2.92(2.79) | 7.55(7.02) | United States |
| Pimavanserin tartrate | 371 | 5.21(4.70, 5.78) | 5.17(4.69, 5.70) | 2.35(2.20) | 5.09(4.67) | United States |
| Fingolimod hydrochloride | 367 | 7.56(6.81, 8.39) | 7.47(6.77, 8.24) | 2.88(2.73) | 7.35(6.74) | United States |
| Dalfampridine | 356 | 3.61(3.25, 4.02) | 3.6(03.26, 3.97) | 1.83(1.68) | 3.55(3.25) | United States |
| Pregabalin | 302 | 2.34(2.08, 2.62) | 2.33(2.07, 2.62) | 1.21(1.04) | 2.31(2.10) | United States |
| Ocrelizumab | 241 | 4.12(3.62, 4.68) | 4.09(3.64, 4.60) | 2.02(1.84) | 4.06(3.65) | United States |
| Topiramate | 224 | 9.13(7.99, 10.42) | 8.99(7.84, 10.31) | 3.15(2.96) | 8.90(7.96) | United States |
| Gabapentin | 188 | 2.49(2.16, 2.88) | 2.48(2.16, 2.84) | 1.30(1.10) | 2.47(2.19) | United States |
| Diroximel fumarate | 186 | 11.49(9.93, 13.29) | 11.26(9.82, 12.92) | 3.48(3.27) | 11.17(9.89) | United States |
| Avapritinib | 180 | 12.85(11.08, 14.91) | 12.57(10.96, 14.42) | 3.64(3.43) | 12.47(11.01) | United States |
| Glatiramer acetate | 173 | 2.58(2.22, 3) | 2.57(2.2, 3.01) | 1.36(1.14) | 2.56(2.26) | United States |
| Oxcarbazepine | 158 | 30.70(26.13, 36.06) | 29.05(24.83, 33.98) | 4.85(4.62) | 28.84(25.20) | United States |
| Ofatumumab | 154 | 3.94(3.36, 4.63) | 3.92(3.35, 4.59) | 1.96(1.74) | 3.90(3.41) | United States |
| Duloxetine | 135 | 2.87(2.42, 3.40) | 2.86(2.40, 3.41) | 1.51(1.26) | 2.84(2.47) | United States |
| Divalproex sodium | 132 | 14.27(12.00, 16.97) | 13.92(11.67, 16.61) | 3.79(3.54) | 13.83(11.96) | United States |
| Clonazepam | 131 | 7.23(6.08, 8.60) | 7.15(5.99, 8.53) | 2.83(2.58) | 7.11(6.15) | United States |
| Peginterferon beta-1a | 130 | 7.85(6.60, 9.34) | 7.75(6.50, 9.25) | 2.95(2.70) | 7.71(6.66) | United States |
| Niraparib | 124 | 4.04(3.39, 4.83) | 4.02(3.37, 4.80) | 2.00(1.75) | 4.00(3.45) | United States |
| Levetiracetam | 109 | 3.51(2.91, 4.24) | 3.49(2.87, 4.25) | 1.8(1.53) | 3.48(2.97) | United States |
| Lamotrigine | 83 | 3.01(2.43, 3.74) | 3.00(2.42, 3.72) | 1.58(1.27) | 2.99(2.50) | United States |
| Ciprofloxacin | 78 | 3.17(2.54, 3.96) | 3.16(2.55, 3.92) | 1.65(1.34) | 3.15(2.61) | United States |
| Quetiapine | 75 | 3.26(2.59, 4.09) | 3.24(2.56, 4.10) | 1.69(1.37) | 3.24(2.67) | United States |
| Alemtuzumab | 72 | 7.34(5.81, 9.26) | 7.25(5.73, 9.17) | 2.85(2.52) | 7.23(5.95) | United States |
| Olanzapine | 62 | 2.88(2.25, 3.70) | 2.87(2.22, 3.70) | 1.52(1.16) | 2.87(2.33) | United States |
| Carbidopa/Levodopa | 59 | 4.87(3.77, 6.30) | 4.84(3.75, 6.24) | 2.27(1.90) | 4.82(3.89) | United States |
| Diltiazem | 111 | 47.80(38.88, 58.75) | 40.57(34.01, 48.40) | 5.28(4.98) | 38.76(32.61) | Canada |
| Amlodipine besylate | 110 | 9.52(7.83, 11.56) | 9.22(7.58, 11.22) | 3.14(2.87) | 8.84(7.51) | Canada |
| Nitroglycerin | 77 | 53.14(41.44, 68.14) | 44.24(35.66, 54.88) | 5.42(5.07) | 42.86(34.81) | Canada |
| Carbamazepine | 75 | 25.57(20.1, 32.53) | 23.35(18.82, 28.97) | 4.5(4.16) | 22.66(18.53) | Canada |
| Furosemide | 65 | 22.04(17.05, 28.48) | 20.37(16.1, 25.77) | 4.31(3.95) | 19.85(16.02) | Canada |
| Omeprazole sodium | 48 | 20.02(14.88, 26.93) | 18.64(14.17, 24.53) | 4.19(3.77) | 18.29(14.27) | Canada |
| Gabapentin | 48 | 3.72(2.79, 4.96) | 3.68(2.8, 4.84) | 1.86(1.45) | 3.63(2.85) | Canada |
| Topiramate | 37 | 8.49(6.1, 11.81) | 8.24(6.02, 11.28) | 3.02(2.55) | 8.13(6.17) | Canada |
| Tiotropium bromide monohydrate | 32 | 4.82(3.39, 6.85) | 4.75(3.34, 6.76) | 2.23(1.73) | 4.70(3.50) | Canada |
| Morphine sulfate | 31 | 3.89(2.72, 5.56) | 3.85(2.71, 5.48) | 1.93(1.42) | 3.81(2.83) | Canada |
| Rosuvastatin calcium | 29 | 4.49(3.10, 6.50) | 4.43(3.05, 6.43) | 2.13(1.61) | 4.39(3.22) | Canada |
| Carbidopa/Levodopa | 26 | 8.92(6.02, 13.21) | 8.65(5.96, 12.55) | 3.10(2.54) | 8.57(6.16) | Canada |
| Clonazepam | 24 | 3.91(2.61, 5.86) | 3.87(2.61, 5.73) | 1.94(1.37) | 3.84(2.73) | Canada |
| Natalizumab | 23 | 3.57(2.36, 5.39) | 3.53(2.34, 5.33) | 1.81(1.23) | 3.51(2.48) | Canada |
| Fingolimod hydrochloride | 21 | 4.62(3, 7.13) | 4.56(2.96, 7.02) | 2.18(1.57) | 4.53(3.15) | Canada |
| Lorazepam | 18 | 6.59(4.12, 10.55) | 6.45(4.11, 10.12) | 2.68(2.02) | 6.41(4.33) | Canada |
| Duloxetine hydrochloride | 16 | 6.00(3.65, 9.86) | 5.88(3.60, 9.60) | 2.55(1.85) | 5.85(3.86) | Canada |
| Alemtuzumab | 16 | 6.73(4.09, 11.07) | 6.58(4.03, 10.74) | 2.71(2.01) | 6.54(4.31) | Canada |
| Esomeprazole magnesium | 16 | 7.94(4.82, 13.08) | 7.73(4.74, 12.62) | 2.94(2.24) | 7.68(5.06) | Canada |
| Quetiapine fumarate | 9 | 7.12(3.67, 13.83) | 6.95(3.64, 13.27) | 2.79(1.88) | 6.93(3.98) | Canada |
| Diazepam | 9 | 11.89(6.08, 23.22) | 11.39(5.97, 21.75) | 3.51(2.59) | 11.35(6.48) | Canada |
| Amlodipine besylate | 9 | 72.87(34.65, 153.21) | 56.7(32.12, 100.10) | 5.82(4.82) | 56.49(30.33) | Canada |
| Peginterferon alfa-2a | 8 | 5.03(2.49, 10.13) | 4.95(2.49, 9.83) | 2.30(1.35) | 4.93(2.74) | Canada |
| Amitriptyline | 8 | 19.48(9.48, 40.05) | 18.15(9.32, 35.34) | 4.18(3.20) | 18.09(9.90) | Canada |
| Oxycodone hydrochloride | 8 | 4.44(2.20, 8.94) | 4.38(2.21, 8.70) | 2.13(1.17) | 4.37(2.43) | Canada |
| Cetirizine hydrochloride | 8 | 8.50(4.20, 17.22) | 8.26(4.16, 16.40) | 3.04(2.08) | 8.23(4.56) | Canada |
| Duloxetine | 8 | 8.72(4.31, 17.67) | 8.46(4.26, 16.80) | 3.08(2.12) | 8.44(4.67) | Canada |
| Levetiracetam | 7 | 8.01(3.77, 17.03) | 7.80(3.78, 16.11) | 2.96(1.94) | 7.78(4.14) | Canada |
| Olanzapine | 7 | 9.92(4.65, 21.12) | 9.58(4.64, 19.78) | 3.26(2.23) | 9.55(5.07) | Canada |
| Tramadol hydrochloride | 7 | 12.54(5.86, 26.81) | 11.99(5.81, 24.76) | 3.58(2.55) | 11.96(6.33) | Canada |
| Aspirin | 6 | 10.59(4.67, 23.99) | 10.20(4.66, 22.34) | 3.35(2.25) | 10.18(5.13) | Canada |
| Quinapril | 6 | 75.20(30.17, 187.43) | 58.08(28.68, 117.62) | 5.86(4.66) | 57.94(26.98) | Canada |
| Bupropion hydrochloride | 6 | 4.71(2.10, 10.58) | 4.64(2.12, 10.16) | 2.21(1.13) | 4.64(2.36) | Canada |
| Cetirizine hcl | 6 | 7.52(3.33, 16.95) | 7.33(3.35, 16.05) | 2.87(1.78) | 7.31(3.70) | Canada |
| Levetiracetam | 5 | 7.55(3.10, 18.38) | 7.35(3.10, 17.41) | 2.88(1.70) | 7.34(3.48) | Canada |
| Donepezil | 5 | 16.93(6.84, 41.91) | 15.92(6.85, 36.98) | 3.99(2.79) | 15.89(7.44) | Canada |
| Lorlatinib | 5 | 28.47(11.28, 71.87) | 25.67(11.27, 58.47) | 4.68(3.46) | 25.62(11.81) | Canada |
| Nabilone | 5 | 104.41(36.75, 296.59) | 73.99(35.13, 155.82) | 6.21(4.87) | 73.84(30.83) | Canada |
| Dipyridamole | 5 | 89.49(32.21, 248.65) | 66.2(31.43, 139.42) | 6.05(4.73) | 66.07(28.10) | Canada |
| Bevacizumab-bvzr | 5 | 9.64(3.94, 23.56) | 9.32(3.93, 22.08) | 3.22(2.04) | 9.30(4.40) | Canada |
| Galantamine | 4 | 91.08(28.98, 286.24) | 67.06(28.87, 155.77) | 6.07(4.62) | 66.95(25.68) | Canada |
| Risperidone | 4 | 5.86(2.17, 15.80) | 5.75(2.20, 15.02) | 2.52(1.24) | 5.74(2.50) | Canada |
| Sofosbuvir | 4 | 9.36(3.45, 25.42) | 9.06(3.47, 23.67) | 3.18(1.88) | 9.05(3.92) | Canada |
| Mirtazapine | 4 | 8.86(3.27, 24.05) | 8.60(3.29, 22.47) | 3.10(1.81) | 8.58(3.72) | Canada |
| Mepolizumab | 4 | 5.16(1.92, 13.91) | 5.08(1.91, 13.54) | 2.34(1.06) | 5.07(2.21) | Canada |
| Nabilone | 4 | 34.55(12.14, 98.35) | 30.48(12.13, 76.58) | 4.93(3.57) | 30.43(12.68) | Canada |
| Amphetamine aspartate/Amphetamine sulfate/Dextroamphetamine saccharate/Dextroamphetamine sulfate | 4 | 38.53(13.44, 110.49) | 33.53(13.35, 84.24) | 5.07(3.70) | 33.48(13.86) | Canada |
| Aspirin/Dipyridamole | 4 | 40.07(13.94, 115.24) | 34.69(14.08, 85.46) | 5.11(3.75) | 34.63(14.31) | Canada |
| Human immunoglobulin g | 4 | 5.27(1.96, 14.20) | 5.18(1.94, 13.8) | 2.37(1.09) | 5.18(2.26) | Canada |
| Risperidone | 4 | 5.93(2.20, 15.99) | 5.81(2.22, 15.18) | 2.54(1.25) | 5.8(2.53) | Canada |
| Bupropion HCl | 4 | 6.63(2.46, 17.92) | 6.49(2.48, 16.96) | 2.70(1.41) | 6.48(2.82) | Canada |
| Lactulose | 4 | 166.98(47.09, 592.11) | 100.59(46.84, 216.04) | 6.65(5.12) | 100.43(34.82) | Canada |
| Finasteride | 3 | 15.33(4.77, 49.21) | 14.5(4.84, 43.46) | 3.86(2.39) | 14.48(5.46) | Canada |
| Mirtazapine | 3 | 6.59(2.09, 20.74) | 6.44(2.11, 19.68) | 2.69(1.25) | 6.44(2.47) | Canada |
| Ribavirin | 3 | 7.51(2.38, 23.70) | 7.32(2.40, 22.37) | 2.87(1.43) | 7.31(2.80) | Canada |
| Sertraline hydrochloride | 3 | 8.63(2.73, 27.31) | 8.38(2.74, 25.61) | 3.07(1.62) | 8.37(3.19) | Canada |
| Propofol | 3 | 7.08(2.25, 22.33) | 6.92(2.26, 21.15) | 2.79(1.35) | 6.91(2.64) | Canada |
| Nabilone | 3 | 375.56(62.72, 2248.66) | 150.82(73.03, 311.47) | 7.23(5.41) | 150.64(33.7) | Canada |
| Cyclobenzaprine | 3 | 7.08(2.25, 22.33) | 6.92(2.26, 21.15) | 2.79(1.35) | 6.91(2.64) | Canada |
| Ixekizumab | 3 | 6.77(2.15, 21.31) | 6.61(2.16, 20.2) | 2.72(1.28) | 6.61(2.53) | Canada |
| Amitriptyline HCl | 3 | 150.22(35.88, 628.95) | 94.26(38.26, 232.21) | 6.56(4.86) | 94.15(28.41) | Canada |
| Pramipexole dihydrochloride | 3 | 7.51(2.38, 23.70) | 7.32(2.40 22.37) | 2.87(1.43) | 7.31(2.80) | Canada |
| Lacosamide | 3 | 6.83(2.17, 21.51) | 6.67(2.18, 20.39) | 2.74(1.3) | 6.67(2.55) | Canada |
| Phenylephrine | 3 | 14.17(4.43, 45.38) | 13.47(4.49, 40.37) | 3.75(2.29) | 13.45(5.08) | Canada |
| Haloperidol | 3 | 15.02(4.68, 48.19) | 14.23(4.75, 42.65) | 3.83(2.36) | 14.21(5.36) | Canada |
| Losartan | 3 | 11.21(3.52, 35.66) | 10.77(3.52, 32.92) | 3.43(1.97) | 10.76(4.09) | Canada |
| Amantadine | 3 | 150.22(35.88, 628.95) | 94.26(38.26, 232.21) | 6.56(4.86) | 94.15(28.41) | Canada |
| Cenobamate | 3 | 8.94(2.82, 28.30) | 8.67(2.84, 26.50) | 3.11(1.67) | 8.66(3.30) | Canada |
| Doxepin | 3 | 93.89(24.89, 354.12) | 68.56(26.24, 179.13) | 6.1(4.46) | 68.47(22.55) | Canada |
| Sertraline hydrochloride | 133 | 2.56(2.14, 3.05) | 2.54(2.13, 3.03) | 1.29(1.04) | 2.45(2.12) | United Kingdom |
| Finasteride | 117 | 9.68(8.01, 11.70) | 9.35(7.84, 11.15) | 3.16(2.89) | 8.92(7.62) | United Kingdom |
| Cetirizine HCl | 99 | 11.86(9.65, 14.57) | 11.34(9.32, 13.80) | 3.45(3.15) | 10.9(9.17) | United Kingdom |
| Simvastatin | 96 | 3.64(2.96, 4.47) | 3.60(2.96, 4.38) | 1.80(1.51) | 3.49(2.94) | United Kingdom |
| Levetiracetam | 67 | 3.78(2.96, 4.83) | 3.74(2.96, 4.73) | 1.87(1.52) | 3.66(2.98) | United Kingdom |
| Fluoxetine | 45 | 2.86(2.13, 3.85) | 2.84(2.12, 3.81) | 1.49(1.06) | 2.81(2.19) | United Kingdom |
| Lansoprazole | 44 | 3.78(2.8, 5.11) | 3.74(2.79, 5.02) | 1.88(1.45) | 3.68(2.86) | United Kingdom |
| Topiramate | 42 | 5.16(3.79, 7.02) | 5.07(3.78, 6.8) | 2.32(1.88) | 5.00(3.86) | United Kingdom |
| Bisoprolol fumarate | 41 | 4.47(3.27, 6.11) | 4.40(3.22, 6.02) | 2.12(1.67) | 4.34(3.35) | United Kingdom |
| Carbidopa/Levodopa | 40 | 8.72(6.34, 11.99) | 8.44(6.17, 11.55) | 3.06(2.60) | 8.31(6.37) | United Kingdom |
| Pregabalin | 38 | 3.03(2.19, 4.18) | 3(2.19, 4.11) | 1.57(1.11) | 2.97(2.27) | United Kingdom |
| Lamotrigine | 36 | 2.72(1.95, 3.78) | 2.7(1.93, 3.77) | 1.42(0.95) | 2.67(2.02) | United Kingdom |
| Esomeprazole | 35 | 23.17(16.33, 32.88) | 21.16(15.46, 28.95) | 4.38(3.89) | 20.86(15.56) | United Kingdom |
| Donepezil | 33 | 3.68(2.6, 5.2) | 3.64(2.61, 5.08) | 1.85(1.36) | 3.60(2.70) | United Kingdom |
| Paroxetine | 31 | 2.75(1.93, 3.93) | 2.73(1.92, 3.88) | 1.44(0.93) | 2.71(2.01) | United Kingdom |
| Candesartan | 27 | 9.56(6.49, 14.07) | 9.22(6.35, 13.38) | 3.19(2.64) | 9.12(6.60) | United Kingdom |
| Anastrozole | 24 | 7.05(4.69, 10.60) | 6.87(4.64, 10.17) | 2.77(2.19) | 6.81(4.84) | United Kingdom |
| Natalizumab | 19 | 5.48(3.47, 8.66) | 5.38(3.43, 8.44) | 2.42(1.78) | 5.34(3.65) | United Kingdom |
| Linagliptin | 16 | 11.11(6.71, 18.37) | 10.64(6.52, 17.37) | 3.40(2.70) | 10.58(6.94) | United Kingdom |
| Naproxen | 16 | 12.42(7.5, 20.57) | 11.83(7.39, 18.94) | 3.56(2.85) | 11.76(7.71) | United Kingdom |
| Esomeprazole magnesium | 16 | 15.63(9.4, 25.97) | 14.70(9.18, 23.53) | 3.87(3.16) | 14.61(9.55) | United Kingdom |
| Enzalutamide | 11 | 8.08(4.42, 14.77) | 7.84(4.35, 14.12) | 2.97(2.13) | 7.81(4.72) | United Kingdom |
| Bupivacaine hydrochloride | 9 | 98.54(45.08, 215.38) | 69.28(40.02, 119.94) | 6.11(5.08) | 69.01(35.87) | United Kingdom |
| Valproate sodium | 8 | 4.06(2.01, 8.17) | 4.00(2.01, 7.94) | 2.00(1.04) | 3.99(2.22) | United Kingdom |
| Bortezomib | 7 | 7.05(3.32, 14.97) | 6.87(3.33, 14.19) | 2.78(1.76) | 6.85(3.65) | United Kingdom |
| Rivastigmine | 5 | 12.89(5.23, 31.77) | 12.26(5.18, 29.04) | 3.61(2.42) | 12.24(5.75) | United Kingdom |
| Duloxetine | 5 | 4.41(1.82, 10.70) | 4.35(1.84, 10.30) | 2.12(0.95) | 4.34(2.07) | United Kingdom |
| Siponimod | 5 | 4.30(1.77, 10.42) | 4.24(1.79, 10.04) | 2.08(0.91) | 4.23(2.02) | United Kingdom |
| Leuprolide acetate | 4 | 65.55(21.56, 199.29) | 51.21(21.62, 121.31) | 5.68(4.25) | 51.12(20.16) | United Kingdom |
| Clonazepam | 4 | 7.52(2.78, 20.38) | 7.31(2.8, 19.1) | 2.87(1.58) | 7.30(3.17) | United Kingdom |
| Apomorphine hydrochloride | 4 | 6.85(2.53, 18.53) | 6.68(2.56, 17.45) | 2.74(1.45) | 6.67(2.90) | United Kingdom |
| Exemestane | 4 | 11.33(4.15, 30.94) | 10.84(4.15, 28.32) | 3.44(2.13) | 10.83(4.67) | United Kingdom |
| Modafinil | 4 | 16.39(5.94, 45.23) | 15.36(6.00, 39.35) | 3.94(2.62) | 15.34(6.56) | United Kingdom |
| Tamsulosin HCl | 4 | 4.75(1.76, 12.80) | 4.68(1.76, 12.47) | 2.22(0.94) | 4.67(2.04) | United Kingdom |
| Pravastatin sodium | 3 | 11.86(3.71, 37.88) | 11.33(3.78, 33.96) | 3.5(2.04) | 11.31(4.28) | United Kingdom |
| Epilim chrono | 3 | 20.23(6.21, 65.93) | 18.67(6.35, 54.87) | 4.22(2.73) | 18.65(6.94) | United Kingdom |
| Propranolol hydrochloride | 3 | 8.09(2.56, 25.61) | 7.85(2.57, 23.99) | 2.97(1.52) | 7.84(2.99) | United Kingdom |
| Brivaracetam | 3 | 6.25(1.98, 19.70) | 6.11(2.00, 18.67) | 2.61(1.17) | 6.11(2.34) | United Kingdom |
| Ketamine hydrochloride | 3 | 12.07(3.78, 38.57) | 11.52(3.84, 34.53) | 3.52(2.06) | 11.50(4.35) | United Kingdom |
| Tropicamide | 3 | 13.76(4.29, 44.14) | 13.04(4.35, 39.08) | 3.70(2.24) | 13.02(4.91) | United Kingdom |
| Dutasteride | 3 | 20.85(6.39, 68.02) | 19.19(6.53, 56.40) | 4.26(2.77) | 19.17(7.13) | United Kingdom |
| Valproate sodium | 185 | 34.34(29.24, 40.34) | 31.43(26.87, 36.77) | 4.78(4.55) | 27.46(24.01) | France |
| Carbidopa/Levodopa | 61 | 11.27(8.68, 14.62) | 10.92(8.46, 14.09) | 3.39(3.02) | 10.50(8.44) | France |
| Finasteride | 58 | 52.46(39.56, 69.57) | 45.34(35.84, 57.36) | 5.44(5.04) | 43.52(34.37) | France |
| Lamotrigine | 32 | 4.01(2.82, 5.71) | 3.97(2.79, 5.65) | 1.97(1.47) | 3.91(2.91) | France |
| Valproic acid | 17 | 23.64(14.40, 38.80) | 22.07(13.79, 35.33) | 4.45(3.75) | 21.82(14.41) | France |
| Donepezil | 15 | 28.27(16.63, 48.07) | 26.04(15.95, 42.51) | 4.69(3.95) | 25.77(16.53) | France |
| Carbamazepine | 12 | 4.22(2.38, 7.47) | 4.17(2.36, 7.36) | 2.05(1.26) | 4.15(2.57) | France |
| Carbidopa/Entacapone/Levodopa | 6 | 8.46(3.76, 19.08) | 8.27(3.78, 18.11) | 3.04(1.95) | 8.24(4.17) | France |
| Fesoterodine fumarate | 6 | 14.98(6.6, 34.04) | 14.35(6.55, 31.43) | 3.84(2.74) | 14.29(7.19) | France |
| Acetaminophen/Tramadol hydrochloride | 5 | 4.33(1.79, 10.48) | 4.28(1.81, 10.14) | 2.09(0.93) | 4.27(2.04) | France |
| Epirubicin | 5 | 4.41(1.82, 10.69) | 4.37(1.84, 10.35) | 2.12(0.96) | 4.35(2.08) | France |
| Estradiol | 5 | 30.83(12.29, 77.37) | 28.17(12.13, 65.44) | 4.81(3.60) | 28.07(13.00) | France |
| Pramipexole | 5 | 26.65(10.68, 66.51) | 24.65(10.61, 57.26) | 4.62(3.41) | 24.56(11.43) | France |
| Trospium chloride | 4 | 48.35(16.85, 138.73) | 42.04(16.73, 105.62) | 5.39(4.02) | 41.92(17.36) | France |
| Pemetrexed | 4 | 16.99(6.2, 46.52) | 16.17(6.19, 42.25) | 4.01(2.71) | 16.12(6.94) | France |
| Clobazam | 4 | 5.69(2.11, 15.31) | 5.60(2.1, 14.92) | 2.48(1.20) | 5.59(2.44) | France |
| Lorlatinib | 4 | 10.47(3.86, 28.40) | 10.17(3.89, 26.57) | 3.34(2.05) | 10.14(4.40) | France |
| Metoprolol | 4 | 26.75(9.62, 74.34) | 24.73(9.65, 63.36) | 4.62(3.30) | 24.66(10.48) | France |
| Rivastigmine tartrate | 3 | 9.15(2.90, 28.86) | 8.92(2.92, 27.26) | 3.15(1.71) | 8.90(3.40) | France |
| Entacapone | 3 | 55.43(16.22, 189.34) | 47.26(16.72, 133.55) | 5.56(4.02) | 47.16(16.87) | France |
| Onabotulinumtoxina | 3 | 6.54(2.08, 20.55) | 6.43(2.10, 19.65) | 2.68(1.25) | 6.42(2.46) | France |
| Epirubicin hydrochloride | 3 | 10.83(3.42, 34.27) | 10.5(3.44, 32.09) | 3.39(1.94) | 10.48(4.00) | France |
| Sofosbuvir/Velpatasvir | 3 | 9.92(3.14, 31.33) | 9.64(3.15, 29.46) | 3.27(1.82) | 9.63(3.68) | France |
| Dronabinol | 3 | 7.19(2.29, 22.61) | 7.05(2.31, 21.55) | 2.82(1.38) | 7.04(2.70) | France |
| Desloratadine | 3 | 10.02(3.17, 31.67) | 9.74(3.19, 29.77) | 3.28(1.84) | 9.72(3.71) | France |
| Nitrous oxide | 3 | 10.71(3.38, 33.87) | 10.39(3.40, 31.75) | 3.37(1.93) | 10.37(3.95) | France |
| Sevoflurane | 3 | 9.06(2.87, 28.58) | 8.83(2.89, 26.99) | 3.14(1.70) | 8.82(3.37) | France |
| Levetiracetam | 53 | 3.77(2.85, 4.98) | 3.74(2.84, 4.92) | 1.85(1.45) | 3.60(2.85) | Germany |
| Carbidopa/Levodopa | 43 | 3.00(2.21, 4.08) | 2.98(2.22, 4.00) | 1.54(1.10) | 2.90(2.24) | Germany |
| Ocrelizumab | 42 | 3.23(2.37, 4.41) | 3.21(2.35, 4.39) | 1.64(1.20) | 3.12(2.41) | Germany |
| Citalopram hydrobromide | 32 | 2.95(2.07, 4.20) | 2.93(2.06, 4.17) | 1.52(1.02) | 2.87(2.14) | Germany |
| Finasteride | 22 | 21.21(13.71, 32.82) | 19.91(13.19, 30.05) | 4.29(3.67) | 19.52(13.55) | Germany |
| Topiramate | 18 | 11.62(7.23, 18.67) | 11.23(7.15, 17.63) | 3.47(2.80) | 11.06(7.43) | Germany |
| Ciprofloxacin | 17 | 3.05(1.88, 4.93) | 3.03(1.89, 4.85) | 1.58(0.91) | 3.00(2.00) | Germany |
| Tamsulosin | 16 | 8.11(4.92, 13.37) | 7.92(4.85, 12.93) | 2.97(2.27) | 7.82(5.14) | Germany |
| Valproate sodium | 14 | 7.49(4.39, 12.77) | 7.33(4.32, 12.44) | 2.86(2.11) | 7.25(4.64) | Germany |
| Lacosamide | 13 | 4.73(2.72, 8.20) | 4.67(2.7, 8.08) | 2.21(1.44) | 4.62(2.92) | Germany |
| Donepezil | 11 | 15.27(8.31, 28.06) | 14.59(8.10, 26.27) | 3.85(3.01) | 14.45(8.68) | Germany |
| Fluoxetine | 10 | 4.93(2.63, 9.24) | 4.87(2.60, 9.12) | 2.27(1.41) | 4.83(2.86) | Germany |
| Lithium | 8 | 55.40(26.02, 117.96) | 47.03(24.63, 89.80) | 5.54(4.52) | 46.68(24.80) | Germany |
| Gabapentin | 8 | 3.72(1.85, 7.49) | 3.69(1.86, 7.33) | 1.87(0.92) | 3.67(2.04) | Germany |
| Epirubicin | 7 | 6.39(3.02, 13.55) | 6.28(2.98, 13.23) | 2.64(1.63) | 6.25(3.33) | Germany |
| Omeprazole magnesium | 7 | 112.16(47.05, 267.37) | 82.23(43.92, 153.96) | 6.35(5.21) | 81.69(39.49) | Germany |
| Quetiapine fumarate | 5 | 4.42(1.83, 10.72) | 4.38(1.81, 10.58) | 2.12(0.96) | 4.36(2.08) | Germany |
| Gadoterate meglumine | 5 | 6.66(2.74, 16.19) | 6.54(2.76, 15.49) | 2.70(1.53) | 6.51(3.10) | Germany |
| Terbinafine hydrochloride | 5 | 37.98(14.96, 96.42) | 33.87(14.87, 77.15) | 5.08(3.84) | 33.71(15.46) | Germany |
| Carbamazepine | 4 | 11.24(4.14, 30.55) | 10.87(4.16, 28.40) | 3.44(2.14) | 10.84(4.69) | Germany |
| Acyclovir | 4 | 4.97(1.85, 13.38) | 4.91(1.84, 13.08) | 2.29(1.01) | 4.89(2.14) | Germany |
| Itraconazole | 4 | 22.48(8.13, 62.19) | 21.00(8.20, 53.8) | 4.39(3.07) | 20.93(8.93) | Germany |
| Galantamine | 4 | 242.86(65.12, 905.67) | 135.37(65.55, 279.56) | 7.08(5.52) | 134.86(44.83) | Germany |
| Rivastigmine tartrate | 4 | 44.97(15.71, 128.75) | 39.30(15.64, 98.73) | 5.29(3.93) | 39.15(16.24) | Germany |
| Voriconazole | 4 | 8.43(3.12, 22.81) | 8.23(3.15, 21.50) | 3.04(1.75) | 8.20(3.57) | Germany |
| Methylprednisolone | 3 | 9.78(3.09, 30.93) | 9.51(3.11, 29.07) | 3.25(1.80) | 9.48(3.62) | Germany |
| Rivastigmine | 3 | 16.24(5.08, 51.98) | 15.47(5.16, 46.36) | 3.95(2.48) | 15.43(5.83) | Germany |
| Diazepam | 3 | 6.94(2.21, 21.84) | 6.81(2.23, 20.81) | 2.76(1.33) | 6.79(2.60) | Germany |
| Ombitasvir/Paritaprevir/Ritonavir | 3 | 7.84(2.49, 24.71) | 7.67(2.51, 23.44) | 2.94(1.50) | 7.65(2.93) | Germany |
| Methylprednisolone | 3 | 6.50(2.07, 20.42) | 6.38(2.09, 19.50) | 2.67(1.23) | 6.37(2.44) | Germany |
| Tapentadol hydrochloride | 3 | 14.91(4.67, 47.60) | 14.26(4.76, 42.74) | 3.83(2.37) | 14.22(5.39) | Germany |
| Interferon beta-1b | 3 | 6.02(1.92, 18.91) | 5.92(1.94, 18.09) | 2.56(1.13) | 5.91(2.27) | Germany |
| Morphine sulfate | 3 | 7.98(2.53, 25.15) | 7.80(2.55, 23.84) | 2.96(1.52) | 7.78(2.98) | Germany |
| Lorazepam | 3 | 8.92(2.82, 28.15) | 8.69(2.84, 26.56) | 3.12(1.67) | 8.67(3.31) | Germany |
