# Supplementary material for: Spectrum and signals of medication-associated cognitive disorder: a comprehensive disproportionality analysis with cross-database validation
Source: Front Pharmacol. 2026 Apr 10;17:1762761. doi: 10.3389/fphar.2026.1762761 (PMC13106381; doi:10.3389/fphar.2026.1762761)
Supplement: Supplementary file 6 [file Table4.docx]

**Table S4** Comparison of Baseline Traits and Medication Use in Patients With and Without Cognitive Disorder

| Variables | Total (n = 2824986) | Non-cognitive disorder  (n = 2817131) | Cognitive disorder  (n = 7855) | p |
| --- | --- | --- | --- | --- |
| Age, Median (Q1, Q3) | 58.00(42, 70) | 58.25(42.00, 70.00) | 55.00(41.00, 69.00) | < 0.001 |
| Sex, n (%) |  |  |  | 0.006 |
| Female | 1686463(59.70) | 1681723(59.70) | 4740(60.34) |  |
| Male | 1121602 39.70) | 1118513(39.70) | 3089(39.33) |  |
| Weight, Median (Q1, Q3) | 72.57(59.86, 88.00) | 72.57(59.86, 88.00) | 72.11(60.00, 86.00) | 0.372 |
| Reporter, n (%) |  |  |  | < 0.001 |
| Consumer | 1078224(38.17) | 1074663(38.15) | 3561(45.33) |  |
| Lawyer | 34017(1.20) | 33887(1.20) | 130(1.65) |  |
| Other health-professional | 343287(12.15) | 342432(12.16) | 855(10.88) |  |
| Pharmacist | 428569(15.17) | 427744(15.18) | 825(10.50) |  |
| Physician | 739458(26.18) | 737587(26.18) | 1871(23.82) |  |
| Registered Nurse | 2606(0.09) | 2595(0.09) | 11(0.14) |  |
| Unknown indication, n (%) |  |  |  | < 0.001 |
| No | 2457569(86.99) | 2451048(87.01) | 6521(83.02) |  |
| Yes | 367417(13.01) | 366083(12.99) | 1334(16.98) |  |
| Rheumatoid arthritis, n (%) |  |  |  | < 0.001 |
| No | 2730364(96.65) | 2722596(96.64) | 7768(98.89) |  |
| Yes | 94622(3.35) | 94535(3.36) | 87(1.11) |  |
| Hypertension, n (%) |  |  |  | < 0.001 |
| No | 2703281(95.69) | 2695599(95.69) | 7682(97.80) |  |
| Yes | 121705(4.31) | 121532(4.31) | 173(2.20) |  |
| Myeloma, n (%) |  |  |  | < 0.001 |
| No | 2773079(98.16) | 2765283(98.16) | 7796(99.25) |  |
| Yes | 51907(1.84) | 51848(1.84) | 59(0.75) |  |
| Gastroesophageal reflux disease, n (%) |  |  |  | < 0.001 |
| No | 2802888(99.22) | 2795064(99.22) | 7824(99.61) |  |
| Yes | 22098(0.78) | 22067(0.78) | 31(0.39) |  |
| Pain, n (%) |  |  |  | 0.196 |
| No | 2749790(97.34) | 2742163(97.34) | 7627(97.10) |  |
| Yes | 75196(2.66) | 74968(2.66) | 228(2.90) |  |
| Diabetes mellitus, n (%) |  |  |  | < 0.001 |
| No | 2724816(96.45) | 2717063(96.45) | 7753(98.70) |  |
| Yes | 100170(3.55) | 100068(3.55) | 102(1.30) |  |
| Depression, n (%) |  |  |  | < 0.001 |
| No | 2775203(98.24) | 2767677(98.24) | 7526(95.81) |  |
| Yes | 49783(1.76) | 49454(1.76) | 329(4.19) |  |
| Breast cancer, n (%) |  |  |  | 0.008 |
| No | 2763947(97.84) | 2756227(97.84) | 7720(98.28) |  |
| Yes | 61039(2.16) | 60904(2.16) | 135(1.72) |  |
| Crohn’s disease, n (%) |  |  |  | < 0.001 |
| No | 2782020(98.48) | 2774186(98.48) | 7834(99.73) |  |
| Yes | 42966(1.52) | 42945(1.52) | 21(0.27) |  |
| Pulmonary arterial hypertension, n (%) |  |  |  | < 0.001 |
| No | 2793138(98.87) | 2785297(98.87) | 7841(99.82) |  |
| Yes | 31848(1.13) | 31834(1.13) | 14(0.18) |  |
| Atrial fibrillation, n (%) |  |  |  | 0.003 |
| No | 2796403(98.99) | 2788601(98.99) | 7802(99.33) |  |
| Yes | 28583(1.01) | 28530(1.01) | 53(0.67) |  |
| Asthma, n (%) |  |  |  | 0.002 |
| No | 2796928(99.01) | 2789123(99.01) | 7805(99.36) |  |
| Yes | 28058(0.99) | 28008(0.99) | 50(0.64) |  |
| Anxiety, n (%) |  |  |  | < 0.001 |
| No | 2801357(99.16) | 2793693(99.17) | 7664(97.57) |  |
| Yes | 23629(0.84) | 23438(0.83) | 191(2.43) |  |
| Contraception, n (%) |  |  |  | < 0.001 |
| No | 2735375(96.83) | 2727589(96.82) | 7786(99.12) |  |
| Yes | 89611(3.17) | 89542(3.18) | 69(0.88) |  |
| Psoriasis, n (%) |  |  |  | < 0.001 |
| No | 2792906(98.86) | 2785066(98.86) | 7840(99.81) |  |
| Yes | 32080(1.14) | 32065(1.14) | 15(0.19) |  |
| Chronic obstructive pulmonary disease, n (%) |  |  |  | < 0.001 |
| No | 2807017(99.36) | 2799170(99.36) | 7847(99.90) |  |
| Yes | 17969(0.64) | 17961(0.64) | 8(0.10) |  |
| Psoriatic arthropathy, n (%) |  |  |  | < 0.001 |
| No | 2806819(99.36) | 2798979(99.36) | 7840(99.81) |  |
| Yes | 18167(0.64) | 18152(0.64) | 15(0.19) |  |
| Constipation, n (%) |  |  |  | 0.401 |
| No | 2814552(99.63) | 2806721(99.63) | 7831(99.69) |  |
| Yes | 10434(0.37) | 10410(0.37) | 24(0.31) |  |
| Multiple sclerosis, n (%) |  |  |  | < 0.001 |
| No | 2782485(98.50) | 2775070(98.51) | 7415(94.40) |  |
| Yes | 42501(1.50) | 42061(1.49) | 440(5.60) |  |
| Natalizumab, n (%) |  |  |  | < 0.001 |
| No | 2819309(99.80) | 2811530(99.80) | 7779(99.03) |  |
| Yes | 5677(0.20) | 5601(0.20) | 76(0.97) |  |
| Interferon beta. 1a, n (%) |  |  |  | < 0.001 |
| No | 2810627(99.49) | 2802904(99.49) | 7723(98.32) |  |
| Yes | 14359(0.51) | 14227(0.51) | 132(1.68) |  |
| Dimethyl fumarate, n (%) |  |  |  | < 0.001 |
| No | 2820852(99.85) | 2813021(99.85) | 7831(99.69) |  |
| Yes | 4134(0.15) | 4110(0.15) | 24(0.31) |  |
| Finasteride, n (%) |  |  |  | < 0.001 |
| No | 2821664(99.88) | 2814016(99.89) | 7648(97.36) |  |
| Yes | 3322(0.12) | 3115(0.11) | 207(2.64) |  |
| Carbidopa/Levodopa, n (%) |  |  |  | < 0.001 |
| No | 2816756(99.71) | 2809107(99.72) | 7649(97.38) |  |
| Yes | 8230(0.29) | 8024(0.28) | 206(2.62) |  |
| Fingolimod hydrochloride, n (%) |  |  |  | < 0.001 |
| No | 2816401(99.70) | 2808730(99.70) | 7671(97.66) |  |
| Yes | 8585(0.30) | 8401(0.30) | 184(2.34) |  |
| Pregabalin, n (%) |  |  |  | < 0.001 |
| No | 2788983(98.73) | 2781353(98.73) | 7630(97.14) |  |
| Yes | 36003(1.27) | 35778(1.27) | 225(2.86) |  |
| Teriflunomide, n (%) |  |  |  | < 0.001 |
| No | 2822511(99.91) | 2814701(99.91) | 7810(99.43) |  |
| Yes | 2475(0.09) | 2430(0.09) | 45(0.57) |  |
| Valproic acid, n (%) |  |  |  | < 0.001 |
| No | 2823320(99.94) | 2815491(99.94) | 7829(99.67) |  |
| Yes | 1666(0.06) | 1640(0.06) | 26(0.33) |  |
| Topiramate, n (%) |  |  |  | < 0.001 |
| No | 2819071(99.79) | 2811336(99.79) | 7735(98.47) |  |
| Yes | 5915(0.21) | 5795(0.21) | 120(1.53) |  |
| Levetiracetam, n (%) |  |  |  | < 0.001 |
| No | 2817068(99.72) | 2809266(99.72) | 7802(99.33) |  |
| Yes | 7918(0.28) | 7865(0.28) | 53(0.67) |  |
| Gabapentin, n (%) |  |  |  | < 0.001 |
| No | 2811341(99.52) | 2803596(99.52) | 7745(98.60) |  |
| Yes | 13645(0.48) | 13535(0.48) | 110(1.40) |  |
| Dalfampridine, n (%) |  |  |  | < 0.001 |
| No | 2818165(99.76) | 2810385(99.76) | 7780(99.05) |  |
| Yes | 6821(0.24) | 6746(0.24) | 75(0.95) |  |
| pimavanserin tartrate, n (%) |  |  |  | < 0.001 |
| No | 2822990(99.93) | 2815168(99.93) | 7822(99.58) |  |
| Yes | 1996(0.07) | 1963(0.07) | 33(0.42) |  |
| Ocrelizumab, n (%) |  |  |  | < 0.001 |
| No | 2815084(99.65) | 2807333(99.65) | 7751(98.68) |  |
| Yes | 9902(0.35) | 9798(0.35) | 104(1.32) |  |
| Lamotrigine, n (%) |  |  |  | < 0.001 |
| No | 2817405(99.73) | 2809621(99.73) | 7784(99.10) |  |
| Yes | 7581(0.27) | 7510(0.27) | 71(0.90) |  |
| Escitalopram oxalate, n (%) |  |  |  | < 0.001 |
| No | 2814406(99.63) | 2806645(99.63) | 7761(98.80) |  |
| Yes | 10580(0.37) | 10486(0.37) | 94(1.20) |  |
| Olanzapine, n (%) |  |  |  | < 0.001 |
| No | 2816507(99.70) | 2808711(99.70) | 7796(99.25) |  |
| Yes | 8479(0.30) | 8420(0.30) | 59(0.75) |  |
| Sertraline hydrochloride, n (%) |  |  |  | < 0.001 |
| No | 2810122(99.47) | 2802388(99.48) | 7734(98.46) |  |
| Yes | 14864(0.53) | 14743(0.52) | 121(1.54) |  |
| Venlafaxine hydrochloride, n (%) |  |  |  | < 0.001 |
| No | 2811921(99.54) | 2804150(99.54) | 7771(98.93) |  |
| Yes | 13065(0.46) | 12981(0.46) | 84(1.07) |  |
| Duloxetine hydrochloride, n (%) |  |  |  | < 0.001 |
| No | 2818328(99.76) | 2810539(99.77) | 7789(99.16) |  |
| Yes | 6658(0.24) | 6592(0.23) | 66(0.84) |  |
| Carbamazepine, n (%) |  |  |  | < 0.001 |
| No | 2821782(99.89) | 2813949(99.89) | 7833(99.72) |  |
| Yes | 3204(0.11) | 3182(0.11) | 22(0.28) |  |
| Clonazepam, n (%) |  |  |  | < 0.001 |
| No | 2821955(99.89) | 2814204(99.90) | 7751(98.68) |  |
| Yes | 3031(0.11) | 2927(0.10) | 104(1.32) |  |
| Viprofloxacin, n (%) |  |  |  | < 0.001 |
| No | 2812121(99.54) | 2804392(99.55) | 7729(98.40) |  |
| Yes | 12865(0.46) | 12739(0.45) | 126(1.60) |  |
| Avapritinib, n (%) |  |  |  | < 0.001 |
| No | 2824819(99.99) | 2816976(99.99) | 7843(99.85) |  |
| Yes | 167(0.01) | 155(0.01) | 12(0.15) |  |
| Diroximel fumarate, n (%) |  |  |  | 0.049 |
| No | 2824859(100.00) | 2817006(100.00) | 7853(99.97) |  |
| Yes | 127(0.00) | 125(0.00) | 2(0.03) |  |
| haloperidol, n (%) |  |  |  | < 0.001 |
| No | 2822338(99.91) | 2814590(99.91) | 7748(98.64) |  |
| Yes | 2648(0.09) | 2541(0.09) | 107(1.36) |  |
| Rivastigmine tartrate, n (%) |  |  |  | < 0.001 |
| No | 2823253(99.94) | 2815428(99.94) | 7825(99.62) |  |
| Yes | 1733(0.06) | 1703(0.06) | 30(0.38) |  |
| ofatumumab, n (%) |  |  |  | 0.639 |
| No | 2823357(99.94) | 2815505(99.94) | 7852(99.96) |  |
| Yes | 1629(0.06) | 1626(0.06) | 3(0.04) |  |
| Donepezil hydrochloride, n (%) |  |  |  | < 0.001 |
| No | 2823045(99.93) | 2815240(99.93) | 7805(99.36) |  |
| Yes | 1941(0.07) | 1891(0.07) | 50(0.64) |  |
