# Supplementary material for: Spectrum and signals of medication-associated cognitive disorder: a comprehensive disproportionality analysis with cross-database validation
Source: Front Pharmacol. 2026 Apr 10;17:1762761. doi: 10.3389/fphar.2026.1762761 (PMC13106381; doi:10.3389/fphar.2026.1762761)
Supplement: Supplementary file 7 [file Table6.docx]

**Table S6** The foremost 50 drugs that cause cognitive disorder, memory impairment, confusional state and disturbance in attention

| **Drug** | **Case Reports** | **ROR (95% CI)** | **PRR (95% CI)** | **IC (IC025)** | **EBGM (EBGM05)** | **Group** |
| --- | --- | --- | --- | --- | --- | --- |
| Natalizumab | 3254 | 9.48 (9.15, 9.83) | 9.32 (8.96, 9.69) | 3.12 (3.06) | 8.67 (8.41) | Cognitive disorder |
| Interferon beta-1a | 1984 | 5.99 (5.72, 6.27) | 5.93 (5.70, 6.17) | 2.51 (2.44) | 5.69 (5.48) | Cognitive disorder |
| Dimethyl | 1440 | 6.21 (5.89, 6.55) | 6.14 (5.79, 6.51) | 2.58 (2.50) | 5.96 (5.70) | Cognitive disorder |
| Finasteride | 1370 | 54.09 (51.10, 57.25) | 48.52 (45.75, 51.46) | 5.55 (5.47) | 46.96 (44.78) | Cognitive disorder |
| Carbidopa/Levodopa | 1245 | 10.94 (10.33, 11.58) | 10.71 (10.10, 11.36) | 3.38 (3.30) | 10.42 (9.93) | Cognitive disorder |
| Fingolimod | 1001 | 5.79 (5.43, 6.16) | 5.73 (5.40, 6.08) | 2.49 (2.40) | 5.61 (5.32) | Cognitive disorder |
| Pregabalin | 614 | 2.39 (2.21, 2.59) | 2.39 (2.21, 2.58) | 1.24 (1.13) | 2.37 (2.21) | Cognitive disorder |
| Teriflunomide | 531 | 6.19 (5.68, 6.75) | 6.12 (5.66, 6.62) | 2.60 (2.47) | 6.06 (5.64) | Cognitive disorder |
| Valproate sodium | 511 | 11.26 (10.31, 12.30) | 11.01 (10.18, 11.91) | 3.45 (3.32) | 10.89 (10.12) | Cognitive disorder |
| Topiramate | 488 | 8.29 (7.57, 9.07) | 8.15 (7.39, 8.99) | 3.01 (2.88) | 8.07 (7.49) | Cognitive disorder |
| Levetiracetam | 471 | 4.03 (3.68, 4.41) | 4.00 (3.63, 4.41) | 1.99 (1.86) | 3.97 (3.68) | Cognitive disorder |
| Gabapentin | 434 | 2.94 (2.67, 3.23) | 2.92 (2.65, 3.22) | 1.54 (1.40) | 2.90 (2.68) | Cognitive disorder |
| Dalfampridine | 406 | 3.12 (2.83, 3.44) | 3.11 (2.82, 3.43) | 1.63 (1.48) | 3.08 (2.84) | Cognitive disorder |
| Pimavanserin | 373 | 4.47 (4.03, 4.95) | 4.43 (4.02, 4.89) | 2.14 (1.99) | 4.40 (4.04) | Cognitive disorder |
| Ocrelizumab | 356 | 3.16 (2.85, 3.51) | 3.14 (2.85, 3.46) | 1.64 (1.49) | 3.13 (2.86) | Cognitive disorder |
| Lamotrigine | 341 | 2.95 (2.65, 3.28) | 2.94 (2.67, 3.24) | 1.55 (1.39) | 2.92 (2.67) | Cognitive disorder |
| Escitalopram | 327 | 3.11 (2.79, 3.47) | 3.09 (2.75, 3.48) | 1.62 (1.46) | 3.08 (2.81) | Cognitive disorder |
| Olanzapine | 326 | 2.96 (2.66, 3.31) | 2.95 (2.62, 3.32) | 1.55 (1.40) | 2.94 (2.68) | Cognitive disorder |
| Sertraline | 295 | 2.56 (2.28, 2.87) | 2.55 (2.27, 2.87) | 1.34 (1.18) | 2.54 (2.31) | Cognitive disorder |
| Venlafaxine | 265 | 2.43 (2.15, 2.74) | 2.42 (2.15, 2.72) | 1.27 (1.09) | 2.41 (2.18) | Cognitive disorder |
| Duloxetine | 252 | 2.41 (2.13, 2.73) | 2.40 (2.13, 2.7) | 1.26 (1.08) | 2.39 (2.16) | Cognitive disorder |
| Carbamazepine | 234 | 4.97 (4.36, 5.65) | 4.92 (4.29, 5.64) | 2.29 (2.11) | 4.90 (4.40) | Cognitive disorder |
| Clonazepam | 224 | 5.17 (4.53, 5.90) | 5.12 (4.46, 5.87) | 2.35 (2.16) | 5.10 (4.56) | Cognitive disorder |
| Divalproex sodium | 210 | 7.45 (6.50, 8.54) | 7.35 (6.41, 8.43) | 2.87 (2.67) | 7.31 (6.52) | Cognitive disorder |
| Ciprofloxacin | 208 | 2.40 (2.09, 2.75) | 2.39 (2.08, 2.74) | 1.25 (1.06) | 2.38 (2.13) | Cognitive disorder |
| Avapritinib | 192 | 10.80 (9.36, 12.47) | 10.57 (9.21, 12.12) | 3.40 (3.19) | 10.53 (9.34) | Cognitive disorder |
| Diroximel | 186 | 9.64 (8.33, 11.15) | 9.46 (8.25, 10.85) | 3.24 (3.03) | 9.42 (8.34) | Cognitive disorder |
| Haloperidol | 180 | 6.79 (5.86, 7.87) | 6.70 (5.84, 7.69) | 2.74 (2.53) | 6.68 (5.90) | Cognitive disorder |
| Rivastigmine | 177 | 5.51 (4.75, 6.40) | 5.46 (4.76, 6.26) | 2.44 (2.23) | 5.44 (4.81) | Cognitive disorder |
| Ofatumumab | 174 | 2.95 (2.54, 3.42) | 2.94 (2.51, 3.44) | 1.55 (1.33) | 2.93 (2.58) | Cognitive disorder |
| Donepezil | 162 | 8.10 (6.93, 9.47) | 7.98 (6.82, 9.33) | 2.99 (2.77) | 7.95 (6.98) | Cognitive disorder |
| Oxcarbazepine | 151 | 31.34 (26.57, 36.98) | 29.36 (25.1, 34.34) | 4.87 (4.63) | 29.26 (25.48) | Cognitive disorder |
| Fluoxetine | 141 | 2.48 (2.10, 2.93) | 2.48 (2.12, 2.90) | 1.30 (1.07) | 2.47 (2.15) | Cognitive disorder |
| Niraparib | 129 | 2.86 (2.40, 3.40) | 2.84 (2.38, 3.39) | 1.51 (1.26) | 2.84 (2.46) | Cognitive disorder |
| Interferon beta-1b | 128 | 2.43 (2.04, 2.89) | 2.42 (2.03, 2.89) | 1.27 (1.02) | 2.42 (2.09) | Cognitive disorder |
| Alemtuzumab | 116 | 4.59 (3.82, 5.51) | 4.55 (3.81, 5.43) | 2.18 (1.92) | 4.54 (3.90) | Cognitive disorder |
| Lorlatinib | 115 | 13.56 (11.26, 16.32) | 13.19 (11.06, 15.73) | 3.72 (3.45) | 13.15 (11.26) | Cognitive disorder |
| Mirtazapine | 109 | 2.62 (2.17, 3.17) | 2.61 (2.15, 3.18) | 1.38 (1.11) | 2.61 (2.23) | Cognitive disorder |
| Phenytoin | 99 | 2.70 (2.22, 3.30) | 2.69 (2.21, 3.27) | 1.43 (1.14) | 2.69 (2.28) | Cognitive disorder |
| Siponimod | 81 | 4.63 (3.72, 5.76) | 4.59 (3.70, 5.69) | 2.20 (1.88) | 4.59 (3.82) | Cognitive disorder |
| Nitroglycerin | 78 | 9.08 (7.25, 11.36) | 8.92 (7.19, 11.07) | 3.15 (2.83) | 8.90 (7.38) | Cognitive disorder |
| Lansoprazole | 47 | 5.09 (3.82, 6.79) | 5.04 (3.76, 6.76) | 2.33 (1.92) | 5.04 (3.96) | Cognitive disorder |
| Zolpidem | 46 | 2.71 (2.03, 3.62) | 2.70 (2.01, 3.62) | 1.43 (1.02) | 2.70 (2.11) | Cognitive disorder |
| Ixazomib | 46 | 2.76 (2.06, 3.68) | 2.74 (2.04, 3.68) | 1.46 (1.04) | 2.74 (2.15) | Cognitive disorder |
| Lorazepam | 45 | 3.88 (2.89, 5.21) | 3.86 (2.88, 5.18) | 1.95 (1.53) | 3.85 (3.02) | Cognitive disorder |
| Lacosamide | 44 | 2.69 (2.00, 3.62) | 2.68 (2.00, 3.60) | 1.42 (1.00) | 2.68 (2.09) | Cognitive disorder |
| Diltiazem | 44 | 12.53 (9.28, 16.91) | 12.21 (9.10, 16.38) | 3.61 (3.18) | 12.2 (9.50) | Cognitive disorder |
| Oxycodone | 41 | 3.43 (2.52, 4.66) | 3.41 (2.49, 4.67) | 1.77 (1.33) | 3.40 (2.63) | Cognitive disorder |
| Tramadol | 40 | 2.99 (2.19, 4.08) | 2.98 (2.18, 4.08) | 1.57 (1.13) | 2.98 (2.29) | Cognitive disorder |
| Paroxetine | 38 | 4.86 (3.53, 6.69) | 4.82 (3.52, 6.60) | 2.27 (1.81) | 4.81 (3.68) | Cognitive disorder |
| Natalizumab | 7445 | 7.23 (7.06, 7.40) | 6.95 (6.82, 7.09) | 2.72 (2.69) | 6.60 (6.47) | Memory impairment |
| Interferon beta-1a | 6250 | 6.44 (6.28, 6.61) | 6.23 (6.11, 6.35) | 2.58 (2.54) | 5.97 (5.84) | Memory impairment |
| Dimethyl | 4883 | 7.24 (7.03, 7.45) | 6.96 (6.82, 7.10) | 2.75 (2.71) | 6.73 (6.56) | Memory impairment |
| Pregabalin | 2891 | 3.84 (3.70, 3.99) | 3.77 (3.63, 3.92) | 1.89 (1.84) | 3.71 (3.59) | Memory impairment |
| Valsartan | 2839 | 2.93 (2.82, 3.04) | 2.89 (2.78, 3.01) | 1.51 (1.46) | 2.85 (2.76) | Memory impairment |
| Fingolimod | 2746 | 5.57 (5.35, 5.78) | 5.40 (5.19, 5.62) | 2.41 (2.35) | 5.31 (5.14) | Memory impairment |
| Tofacitinib | 2529 | 3.02 (2.90, 3.14) | 2.98 (2.87, 3.10) | 1.55 (1.50) | 2.94 (2.84) | Memory impairment |
| Dalfampridine | 1738 | 4.55 (4.33, 4.77) | 4.44 (4.27, 4.62) | 2.14 (2.07) | 4.39 (4.22) | Memory impairment |
| Teriflunomide | 1380 | 5.44 (5.15, 5.74) | 5.28 (4.98, 5.60) | 2.39 (2.31) | 5.24 (5.00) | Memory impairment |
| Insulin lispro | 1298 | 2.61 (2.47, 2.75) | 2.58 (2.43, 2.74) | 1.36 (1.28) | 2.56 (2.45) | Memory impairment |
| Sodium oxybate | 1247 | 2.73 (2.58, 2.89) | 2.70 (2.55, 2.86) | 1.42 (1.34) | 2.68 (2.56) | Memory impairment |
| Palbociclib | 1207 | 2.37 (2.24, 2.51) | 2.35 (2.22, 2.49) | 1.22 (1.14) | 2.33 (2.22) | Memory impairment |
| Carbidopa/levodopa | 1202 | 3.29 (3.11, 3.49) | 3.24 (3.05, 3.44) | 1.69 (1.61) | 3.22 (3.07) | Memory impairment |
| Gabapentin | 1107 | 2.50 (2.36, 2.66) | 2.48 (2.34, 2.63) | 1.30 (1.22) | 2.46 (2.34) | Memory impairment |
| Duloxetine | 1036 | 3.35 (3.15, 3.57) | 3.30 (3.11, 3.50) | 1.71 (1.63) | 3.28 (3.12) | Memory impairment |
| Paroxetine | 968 | 3.77 (3.53, 4.02) | 3.70 (3.49, 3.92) | 1.88 (1.79) | 3.68 (3.49) | Memory impairment |
| Ocrelizumab | 863 | 2.56 (2.39, 2.74) | 2.53 (2.39, 2.68) | 1.33 (1.24) | 2.52 (2.38) | Memory impairment |
| Levothyroxine | 799 | 2.66 (2.48, 2.86) | 2.63 (2.43, 2.84) | 1.39 (1.29) | 2.62 (2.47) | Memory impairment |
| Pimavanserin | 746 | 2.98 (2.77, 3.20) | 2.94 (2.72, 3.18) | 1.55 (1.45) | 2.93 (2.76) | Memory impairment |
| Topiramate | 736 | 4.15 (3.86, 4.47) | 4.07 (3.76, 4.40) | 2.02 (1.91) | 4.05 (3.81) | Memory impairment |
| Ofatumumab | 574 | 3.27 (3.01, 3.55) | 3.22 (2.98, 3.48) | 1.68 (1.56) | 3.21 (3.00) | Memory impairment |
| Budesonide/formoterol dihydrate | 539 | 2.83 (2.60, 3.08) | 2.79 (2.58, 3.02) | 1.48 (1.35) | 2.78 (2.59) | Memory impairment |
| Rivastigmine | 526 | 5.56 (5.10, 6.07) | 5.40 (4.99, 5.84) | 2.43 (2.30) | 5.38 (5.00) | Memory impairment |
| Clonazepam | 467 | 3.61 (3.29, 3.95) | 3.54 (3.21, 3.90) | 1.82 (1.69) | 3.54 (3.27) | Memory impairment |
| Zolpidem | 460 | 3.19 (2.90, 3.49) | 3.14 (2.85, 3.46) | 1.65 (1.51) | 3.13 (2.90) | Memory impairment |
| Diroximel | 440 | 7.77 (7.06, 8.55) | 7.43 (6.74, 8.20) | 2.89 (2.75) | 7.41 (6.84) | Memory impairment |
| Insulin human | 437 | 2.27 (2.07, 2.50) | 2.25 (2.04, 2.48) | 1.17 (1.03) | 2.25 (2.08) | Memory impairment |
| Avapritinib | 406 | 7.76 (7.02, 8.58) | 7.43 (6.74, 8.20) | 2.89 (2.74) | 7.40 (6.81) | Memory impairment |
| Interferon beta-1b | 370 | 2.35 (2.12, 2.60) | 2.33 (2.11, 2.57) | 1.22 (1.07) | 2.32 (2.13) | Memory impairment |
| Octreotide | 355 | 2.69 (2.42, 2.99) | 2.66 (2.41, 2.93) | 1.41 (1.26) | 2.66 (2.43) | Memory impairment |
| Interferon alfa-2b | 351 | 3.71 (3.34, 4.13) | 3.65 (3.31, 4.03) | 1.86 (1.71) | 3.64 (3.33) | Memory impairment |
| Niraparib | 346 | 2.56 (2.31, 2.85) | 2.54 (2.30, 2.80) | 1.34 (1.19) | 2.53 (2.32) | Memory impairment |
| Finasteride | 316 | 3.67 (3.29, 4.11) | 3.61 (3.21, 4.06) | 1.85 (1.69) | 3.60 (3.28) | Memory impairment |
| Lacosamide | 299 | 3.06 (2.73, 3.44) | 3.02 (2.68, 3.4) | 1.59 (1.43) | 3.02 (2.74) | Memory impairment |
| Oxcarbazepine | 290 | 4.80 (4.27, 5.40) | 4.68 (4.16, 5.26) | 2.23 (2.06) | 4.68 (4.24) | Memory impairment |
| Alemtuzumab | 281 | 3.73 (3.32, 4.20) | 3.67 (3.26, 4.13) | 1.87 (1.70) | 3.66 (3.31) | Memory impairment |
| Siponimod | 249 | 4.82 (4.24, 5.47) | 4.70 (4.18, 5.29) | 2.23 (2.05) | 4.69 (4.22) | Memory impairment |
| Aclidinium bromide | 187 | 6.41 (5.54, 7.42) | 6.19 (5.40, 7.10) | 2.63 (2.42) | 6.18 (5.47) | Memory impairment |
| Corticotropin | 133 | 2.56 (2.15, 3.04) | 2.53 (2.12, 3.02) | 1.34 (1.09) | 2.53 (2.19) | Memory impairment |
| Cenobamate | 125 | 5.14 (4.30, 6.15) | 5.00 (4.19, 5.96) | 2.32 (2.07) | 5.00 (4.31) | Memory impairment |
| Fesoterodine | 113 | 2.91 (2.41, 3.50) | 2.87 (2.41, 3.42) | 1.52 (1.25) | 2.87 (2.46) | Memory impairment |
| Amphetamine | 109 | 2.45 (2.03, 2.96) | 2.43 (2.00, 2.96) | 1.28 (1.01) | 2.43 (2.07) | Memory impairment |
| Ziconotide | 103 | 7.37 (6.04, 8.98) | 7.07 (5.81, 8.6) | 2.82 (2.54) | 7.06 (5.98) | Memory impairment |
| Sapropterin | 96 | 2.72 (2.22, 3.32) | 2.68 (2.20, 3.26) | 1.42 (1.13) | 2.68 (2.27) | Memory impairment |
| Daclizumab | 95 | 6.26 (5.10, 7.69) | 6.05 (4.97, 7.36) | 2.60 (2.30) | 6.04 (5.09) | Memory impairment |
| Tucatinib | 95 | 4.94 (4.03, 6.06) | 4.81 (3.95, 5.85) | 2.27 (1.97) | 4.81 (4.05) | Memory impairment |
| Boceprevir | 90 | 2.54 (2.06, 3.12) | 2.51 (2.06, 3.05) | 1.33 (1.03) | 2.51 (2.11) | Memory impairment |
| Brivaracetam | 88 | 3.64 (2.95, 4.50) | 3.58 (2.89, 4.44) | 1.84 (1.54) | 3.58 (3.00) | Memory impairment |
| Budesonide/formoterol/glycopyrronium | 80 | 2.86 (2.29, 3.57) | 2.83 (2.28, 3.51) | 1.50 (1.18) | 2.83 (2.35) | Memory impairment |
| Dichlorphenamide | 65 | 6.60 (5.15, 8.46) | 6.36 (5.03, 8.05) | 2.67 (2.31) | 6.36 (5.17) | Memory impairment |
| Pimavanserin | 3286 | 12.16 (11.73, 12.61) | 11.19 (10.76, 11.64) | 3.46 (3.40) | 10.97 (10.64) | Confusional state |
| Duloxetine | 2987 | 8.68 (8.36, 9.01) | 8.19 (7.88, 8.52) | 3.01 (2.95) | 8.05 (7.80) | Confusional state |
| Pregabalin | 2606 | 2.92 (2.80, 3.03) | 2.87 (2.76, 2.98) | 1.51 (1.45) | 2.84 (2.75) | Confusional state |
| Quetiapine | 1876 | 3.12 (2.98, 3.27) | 3.07 (2.95, 3.19) | 1.61 (1.54) | 3.05 (2.93) | Confusional state |
| Olanzapine | 1803 | 4.77 (4.55, 5.00) | 4.64 (4.46, 4.83) | 2.20 (2.13) | 4.59 (4.41) | Confusional state |
| Gabapentin | 1767 | 3.44 (3.28, 3.61) | 3.37 (3.24, 3.50) | 1.74 (1.67) | 3.35 (3.21) | Confusional state |
| Carbidopa/levodopa | 1761 | 4.15 (3.96, 4.35) | 4.05 (3.89, 4.21) | 2.01 (1.94) | 4.01 (3.86) | Confusional state |
| Paroxetine | 1687 | 5.71 (5.43, 5.99) | 5.50 (5.29, 5.72) | 2.45 (2.38) | 5.45 (5.23) | Confusional state |
| Sertraline | 1254 | 3.15 (2.98, 3.33) | 3.10 (2.92, 3.29) | 1.62 (1.54) | 3.08 (2.94) | Confusional state |
| Venlafaxine | 1216 | 3.20 (3.02, 3.39) | 3.14 (2.96, 3.33) | 1.64 (1.56) | 3.13 (2.98) | Confusional state |
| Tramadol | 1166 | 4.71 (4.44, 4.99) | 4.57 (4.31, 4.85) | 2.18 (2.10) | 4.54 (4.33) | Confusional state |
| Ciprofloxacin | 942 | 3.12 (2.92, 3.33) | 3.07 (2.89, 3.26) | 1.61 (1.52) | 3.05 (2.89) | Confusional state |
| Lamotrigine | 916 | 2.25 (2.11, 2.40) | 2.23 (2.10, 2.37) | 1.15 (1.06) | 2.22 (2.10) | Confusional state |
| Zolpidem | 879 | 5.22 (4.88, 5.59) | 5.05 (4.76, 5.36) | 2.33 (2.23) | 5.03 (4.75) | Confusional state |
| Memantine | 852 | 14.46 (13.47, 15.53) | 13.08 (12.33, 13.87) | 3.70 (3.60) | 13.01 (12.26) | Confusional state |
| Levofloxacin | 824 | 2.76 (2.57, 2.95) | 2.72 (2.56, 2.88) | 1.44 (1.34) | 2.71 (2.56) | Confusional state |
| Mirtazapine | 814 | 5.74 (5.35, 6.16) | 5.53 (5.21, 5.86) | 2.46 (2.36) | 5.51 (5.19) | Confusional state |
| Citalopram | 783 | 4.04 (3.76, 4.34) | 3.95 (3.65, 4.27) | 1.97 (1.87) | 3.93 (3.70) | Confusional state |
| Topiramate | 704 | 3.37 (3.12, 3.63) | 3.30 (3.05, 3.57) | 1.72 (1.61) | 3.29 (3.09) | Confusional state |
| Alprazolam | 697 | 2.76 (2.56, 2.98) | 2.73 (2.52, 2.95) | 1.44 (1.33) | 2.72 (2.55) | Confusional state |
| Furosemide | 674 | 3.68 (3.41, 3.97) | 3.60 (3.33, 3.89) | 1.84 (1.73) | 3.59 (3.37) | Confusional state |
| Escitalopram | 625 | 3.62 (3.35, 3.93) | 3.55 (3.28, 3.84) | 1.82 (1.71) | 3.54 (3.31) | Confusional state |
| Rivastigmine | 621 | 6.25 (5.77, 6.78) | 6.01 (5.56, 6.50) | 2.58 (2.46) | 5.98 (5.59) | Confusional state |
| Fluoxetine | 617 | 3.12 (2.88, 3.38) | 3.07 (2.84, 3.32) | 1.61 (1.50) | 3.06 (2.86) | Confusional state |
| Lorazepam | 570 | 4.92 (4.52, 5.35) | 4.77 (4.41, 5.16) | 2.25 (2.13) | 4.76 (4.43) | Confusional state |
| Morphine | 553 | 2.30 (2.11, 2.50) | 2.28 (2.11, 2.47) | 1.18 (1.06) | 2.27 (2.12) | Confusional state |
| Digoxin | 504 | 6.41 (5.86, 7.02) | 6.15 (5.69, 6.65) | 2.62 (2.49) | 6.13 (5.69) | Confusional state |
| Rifaximin | 491 | 4.95 (4.52, 5.42) | 4.80 (4.44, 5.19) | 2.26 (2.13) | 4.79 (4.44) | Confusional state |
| Ramipril | 479 | 3.55 (3.24, 3.89) | 3.48 (3.16, 3.84) | 1.80 (1.67) | 3.47 (3.22) | Confusional state |
| Carbamazepine | 472 | 2.84 (2.59, 3.11) | 2.80 (2.54, 3.09) | 1.48 (1.35) | 2.79 (2.59) | Confusional state |
| Droxidopa | 406 | 2.67 (2.42, 2.95) | 2.64 (2.39, 2.91) | 1.40 (1.25) | 2.63 (2.42) | Confusional state |
| Sorafenib | 391 | 2.67 (2.41, 2.95) | 2.63 (2.38, 2.90) | 1.39 (1.25) | 2.63 (2.41) | Confusional state |
| Moxifloxacin | 367 | 3.15 (2.84, 3.49) | 3.09 (2.80, 3.41) | 1.63 (1.48) | 3.09 (2.83) | Confusional state |
| Oseltamivir | 364 | 3.14 (2.82, 3.48) | 3.08 (2.79, 3.4) | 1.62 (1.47) | 3.08 (2.82) | Confusional state |
| Pantoprazole | 357 | 2.34 (2.11, 2.60) | 2.32 (2.10, 2.56) | 1.21 (1.06) | 2.32 (2.12) | Confusional state |
| Sodium citrate | 285 | 5.63 (5.00, 6.34) | 5.43 (4.83, 6.11) | 2.44 (2.27) | 5.42 (4.91) | Confusional state |
| Octreotide | 280 | 2.29 (2.04, 2.58) | 2.27 (2.02, 2.55) | 1.18 (1.01) | 2.27 (2.06) | Confusional state |
| Baclofen | 214 | 4.78 (4.17, 5.48) | 4.65 (4.05, 5.33) | 2.21 (2.02) | 4.64 (4.14) | Confusional state |
| Leflunomide | 213 | 8.37 (7.29, 9.62) | 7.91 (6.90, 9.07) | 2.98 (2.78) | 7.90 (7.03) | Confusional state |
| Clarithromycin | 207 | 5.22 (4.55, 6.00) | 5.06 (4.41, 5.8) | 2.34 (2.14) | 5.05 (4.50) | Confusional state |
| Axicabtagene ciloleucel | 197 | 5.21 (4.52, 6.01) | 5.05 (4.40, 5.79) | 2.33 (2.13) | 5.04 (4.47) | Confusional state |
| Fentanyl | 186 | 2.66 (2.30, 3.08) | 2.63 (2.29, 3.02) | 1.39 (1.18) | 2.63 (2.33) | Confusional state |
| Ertapenem | 183 | 10.78 (9.27, 12.54) | 10.01 (8.73, 11.48) | 3.32 (3.11) | 10.00 (8.81) | Confusional state |
| Selinexor | 183 | 5.69 (4.90, 6.59) | 5.48 (4.78, 6.29) | 2.45 (2.24) | 5.48 (4.84) | Confusional state |
| Metronidazole | 181 | 3.71 (3.20, 4.30) | 3.63 (3.16, 4.16) | 1.86 (1.65) | 3.63 (3.21) | Confusional state |
| Interferon alfa-2b | 179 | 4.35 (3.75, 5.05) | 4.24 (3.70, 4.86) | 2.08 (1.87) | 4.23 (3.74) | Confusional state |
| Ziconotide | 178 | 11.84 (10.15, 13.81) | 10.91 (9.51, 12.51) | 3.45 (3.23) | 10.90 (9.58) | Confusional state |
| Valproate sodium | 172 | 3.24 (2.78, 3.77) | 3.18 (2.72, 3.72) | 1.67 (1.45) | 3.18 (2.80) | Confusional state |
| Bisoprolol | 172 | 2.95 (2.54, 3.43) | 2.91 (2.49, 3.40) | 1.54 (1.32) | 2.90 (2.56) | Confusional state |
| Amphetamine | 169 | 3.26 (2.80, 3.80) | 3.20 (2.74, 3.74) | 1.68 (1.46) | 3.20 (2.82) | Confusional state |
| Amphetamine | 1352 | 35.64 (33.69, 37.71) | 32.68 (30.81, 34.66) | 4.99 (4.91) | 31.83 (30.36) | Disturbance in attention |
| Pregabalin | 1347 | 4.44 (4.21, 4.69) | 4.40 (4.15, 4.67) | 2.11 (2.03) | 4.31 (4.12) | Disturbance in attention |
| Paroxetine | 1303 | 13.06 (12.35, 13.82) | 12.66 (11.94, 13.43) | 3.63 (3.55) | 12.36 (11.8) | Disturbance in attention |
| Levothyroxine sodium | 1236 | 10.61 (10.02, 11.23) | 10.34 (9.75, 10.97) | 3.34 (3.26) | 10.11 (9.64) | Disturbance in attention |
| Duloxetine | 1007 | 8.24 (7.74, 8.78) | 8.09 (7.63, 8.58) | 2.99 (2.90) | 7.95 (7.54) | Disturbance in attention |
| Varenicline | 967 | 4.60 (4.31, 4.90) | 4.55 (4.29, 4.83) | 2.17 (2.07) | 4.49 (4.25) | Disturbance in attention |
| Lisdexamfetamine | 921 | 20.69 (19.35, 22.12) | 19.67 (18.55, 20.86) | 4.27 (4.18) | 19.33 (18.27) | Disturbance in attention |
| Methylphenidate | 906 | 20.65 (19.30, 22.09) | 19.63 (18.51, 20.82) | 4.27 (4.17) | 19.29 (18.24) | Disturbance in attention |
| Venlafaxine | 835 | 7.24 (6.76, 7.76) | 7.12 (6.71, 7.55) | 2.81 (2.71) | 7.02 (6.62) | Disturbance in attention |
| Sertraline | 758 | 5.55 (5.16, 5.97) | 5.49 (5.08, 5.94) | 2.44 (2.33) | 5.42 (5.10) | Disturbance in attention |
| Quetiapine | 666 | 3.22 (2.99, 3.48) | 3.20 (2.96, 3.46) | 1.67 (1.56) | 3.18 (2.98) | Disturbance in attention |
| Atomoxetine | 663 | 14.82 (13.71, 16.03) | 14.3 (13.22, 15.47) | 3.82 (3.71) | 14.12 (13.23) | Disturbance in attention |
| Valproic acid | 627 | 7.02 (6.48, 7.60) | 6.91 (6.39, 7.47) | 2.77 (2.66) | 6.83 (6.39) | Disturbance in attention |
| Ciprofloxacin | 592 | 5.76 (5.31, 6.25) | 5.69 (5.26, 6.15) | 2.49 (2.38) | 5.63 (5.26) | Disturbance in attention |
| Gabapentin | 574 | 3.23 (2.98, 3.51) | 3.22 (2.98, 3.48) | 1.67 (1.55) | 3.19 (2.98) | Disturbance in attention |
| Aripiprazole | 574 | 3.10 (2.86, 3.37) | 3.09 (2.86, 3.34) | 1.61 (1.50) | 3.06 (2.86) | Disturbance in attention |
| Topiramate | 547 | 7.72 (7.09, 8.40) | 7.58 (7.01, 8.20) | 2.91 (2.79) | 7.51 (7.00) | Disturbance in attention |
| Montelukast sodium | 539 | 9.20 (8.44, 10.03) | 9.00 (8.32, 9.73) | 3.16 (3.03) | 8.92 (8.30) | Disturbance in attention |
| Bupropion | 506 | 5.14 (4.71, 5.61) | 5.08 (4.70, 5.49) | 2.33 (2.21) | 5.04 (4.68) | Disturbance in attention |
| Lamotrigine | 490 | 3.53 (3.23, 3.86) | 3.51 (3.18, 3.87) | 1.80 (1.67) | 3.49 (3.23) | Disturbance in attention |
| Olanzapine | 452 | 3.47 (3.16, 3.80) | 3.44 (3.12, 3.79) | 1.77 (1.64) | 3.42 (3.17) | Disturbance in attention |
| Sodium oxybate | 446 | 2.41 (2.20, 2.65) | 2.41 (2.19, 2.66) | 1.26 (1.12) | 2.39 (2.21) | Disturbance in attention |
| Finasteride | 440 | 13.03 (11.84, 14.33) | 12.62 (11.44, 13.92) | 3.65 (3.51) | 12.52 (11.56) | Disturbance in attention |
| Levetiracetam | 438 | 3.10 (2.82, 3.41) | 3.08 (2.79, 3.40) | 1.62 (1.48) | 3.06 (2.83) | Disturbance in attention |
| Citalopram | 396 | 5.95 (5.39, 6.58) | 5.88 (5.33, 6.49) | 2.55 (2.40) | 5.84 (5.37) | Disturbance in attention |
| Vortioxetine | 363 | 18.30 (16.46, 20.34) | 17.49 (15.86, 19.29) | 4.12 (3.97) | 17.37 (15.9) | Disturbance in attention |
| Carbidopa/levodopa | 339 | 2.37 (2.13, 2.64) | 2.37 (2.15, 2.61) | 1.24 (1.08) | 2.36 (2.15) | Disturbance in attention |
| Escitalopram | 331 | 5.60 (5.02, 6.25) | 5.53 (5.01, 6.10) | 2.46 (2.30) | 5.50 (5.03) | Disturbance in attention |
| Tramadol | 331 | 3.19 (2.86, 3.56) | 3.17 (2.87, 3.50) | 1.66 (1.50) | 3.16 (2.88) | Disturbance in attention |
| Fluoxetine | 292 | 4.23 (3.77, 4.75) | 4.19 (3.73, 4.71) | 2.06 (1.89) | 4.17 (3.79) | Disturbance in attention |
| Levofloxacin | 281 | 2.73 (2.43, 3.08) | 2.72 (2.42, 3.06) | 1.44 (1.27) | 2.71 (2.46) | Disturbance in attention |
| Cetirizine | 272 | 2.36 (2.09, 2.66) | 2.35 (2.09, 2.64) | 1.23 (1.06) | 2.34 (2.12) | Disturbance in attention |
| Alprazolam | 255 | 2.89 (2.56, 3.27) | 2.88 (2.56, 3.24) | 1.52 (1.34) | 2.87 (2.59) | Disturbance in attention |
| Clonazepam | 251 | 4.81 (4.25, 5.45) | 4.76 (4.23, 5.35) | 2.25 (2.07) | 4.74 (4.27) | Disturbance in attention |
| Desvenlafaxine | 251 | 5.96 (5.26, 6.76) | 5.88 (5.23, 6.61) | 2.55 (2.37) | 5.86 (5.28) | Disturbance in attention |
| Acetaminophen/Hydrocodone bitartrate | 249 | 2.54 (2.25, 2.88) | 2.53 (2.25, 2.85) | 1.34 (1.16) | 2.53 (2.28) | Disturbance in attention |
| Interferon alfa-2a | 203 | 2.41 (2.10, 2.77) | 2.40 (2.09, 2.75) | 1.26 (1.06) | 2.40 (2.14) | Disturbance in attention |
| Mirtazapine | 181 | 3.63 (3.13, 4.20) | 3.60 (3.14, 4.13) | 1.85 (1.64) | 3.60 (3.18) | Disturbance in attention |
| Zolpidem | 158 | 2.67 (2.28, 3.12) | 2.66 (2.27, 3.11) | 1.41 (1.18) | 2.65 (2.33) | Disturbance in attention |
| Carbamazepine | 141 | 2.46 (2.08, 2.90) | 2.45 (2.09, 2.87) | 1.29 (1.05) | 2.45 (2.13) | Disturbance in attention |
| Interferon alfa-2b | 136 | 3.92 (3.31, 4.65) | 3.89 (3.26, 4.64) | 1.96 (1.71) | 3.89 (3.37) | Disturbance in attention |
| Lurasidone | 126 | 3.28 (2.75, 3.91) | 3.26 (2.73, 3.89) | 1.70 (1.45) | 3.25 (2.81) | Disturbance in attention |
| Amiodarone | 108 | 3.42 (2.83, 4.14) | 3.40 (2.79, 4.14) | 1.76 (1.49) | 3.40 (2.90) | Disturbance in attention |
| Lacosamide | 101 | 2.56 (2.10, 3.11) | 2.55 (2.10, 3.10) | 1.35 (1.07) | 2.54 (2.16) | Disturbance in attention |
| Bisoprolol | 95 | 3.11 (2.54, 3.81) | 3.10 (2.55, 3.77) | 1.63 (1.34) | 3.09 (2.61) | Disturbance in attention |
| Acetaminophen | 93 | 2.56 (2.09, 3.14) | 2.55 (2.10, 3.10) | 1.35 (1.06) | 2.55 (2.15) | Disturbance in attention |
| Oxcarbazepine | 86 | 3.49 (2.82, 4.32) | 3.47 (2.80, 4.30) | 1.79 (1.49) | 3.46 (2.90) | Disturbance in attention |
| Brexpiprazole | 77 | 2.66 (2.13, 3.33) | 2.65 (2.14, 3.29) | 1.40 (1.08) | 2.65 (2.19) | Disturbance in attention |
| Mefloquine | 75 | 32.37 (25.56, 40.99) | 29.85 (24.06, 37.03) | 4.90 (4.56) | 29.81 (24.47) | Disturbance in attention |
| Armodafinil | 60 | 4.58 (3.55, 5.91) | 4.54 (3.52, 5.86) | 2.18 (1.82) | 4.53 (3.66) | Disturbance in attention |
